# Identification and Comparative Study of Chemosensory Genes Related to Host Selection by Legs Transcriptome Analysis in the Tea Geometrid *Ectropis obliqua*

**DOI:** 10.1371/journal.pone.0149591

**Published:** 2016-03-01

**Authors:** Long Ma, Zhao-Qun Li, Lei Bian, Xiao-Ming Cai, Zong-Xiu Luo, Yong-Jun Zhang, Zong-Mao Chen

**Affiliations:** 1 Key Laboratory of Tea Biology and Resources Utilization, Ministry of Agriculture, Tea Research Institute, Chinese Academy of Agricultural Sciences, Hangzhou, China; 2 State Key Laboratory for Biology of Plant Diseases and Insect Pests, Institute of Plant Protection, Chinese Academy of Agricultural Sciences, Beijing, China; USDA-ARS, UNITED STATES

## Abstract

Host selection by female moths is fundamental to the survival of their larvae. Detecting and perceiving the non-volatile chemicals of the plant surface involved in gustatory detection determine the host preference. In many lepidopteran species, tarsal chemosensilla are sensitive to non-volatile chemicals and responsible for taste detection. The tea geometrid *Ectropis obliqua* is one devastating chewing pest selectively feeding on limited plants, requiring the specialized sensors to forage certain host for oviposition. In present study, we revealed the distribution of chemosensilla in the ventral side of female fifth tarsomere in *E*. *obliqua*. To investigate its molecular mechanism of gustatory perception, we performed HiSeq 2500 sequencing of the male- and female- legs transcriptome and identified 24 candidate odorant binding proteins (OBPs), 21 chemosensory proteins (CSPs), 2 sensory neuron membrane proteins (SNMPs), 3 gustatory receptors (GRs) and 4 odorant receptors (ORs). Several leg-specific or enriched chemosensory genes were screened by tissue expression analysis, and clustered with functionally validated genes from other moths, suggesting the potential involvement in taste sensation or other physiological processes. The RPKM value analysis revealed that 9 EoblOBPs showed sex discrepancy in the leg expression, 8 being up-regulated in female and only 1 being over expressed in male. These female-biased EoblOBPs indicated an ecological adaption related with host-seeking and oviposition behaviors. Our work will provide basic knowledge for further studies on the molecular mechanism of gustatory perception, and enlighten a host-selection-based control strategy of insect pests.

## Introduction

**Fig 1:**
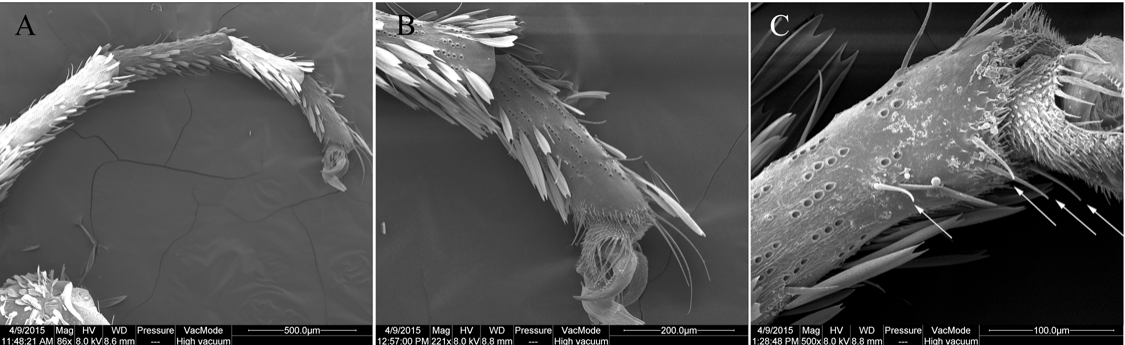
Scanning electron micrographs of foreleg tarsus and chemosensilla of the fifth tarsomere of adult female *E*. *obliqua*. (A) Foreleg tarsus. (B) Magnification of the fifth tarsomere by SEM. (C) Chemosensilla distributed in the ventral side of a female fifth tarsomere.

Herbivorous insects must locate and identify host plants to meet their biological requirements and the demand of reproduction, while the process of host selection for feeding and oviposition involves foraging, landing, contact evaluation and final determination [1]. Olfaction and taste perception play crucial roles in chemical detection and discrimination of the host. As many lepidopteran species are designated to use a limited range of host plants, detecting and perceiving the semiochemicals from the host are particularly important. Generally, insects utilize their sensitive and selective antennae to detect air borne odorant molecules and guide the location from distance, while the subsequent contact evaluation of non-volatile chemicals involved in gustatory detection determines the host preference. Typically, insect contact chemoreceptors, derived from mechanosensory bristles and mainly scattered on the legs, the proboscis, the maxillary and the labial palps, are sensitive to phagostimulants (e.g. sugars, oviposition stimulants and amino acid) [2–5]. Many studies have confirmed the chemosensilla on legs play a principal role in perceiving phytochemical compounds after the insects land on the leaves and start drumming on the surface with the tarsi of prothoracic legs [6–9]. In Papilionidae (such as *Papilio xuthus*, *Heliconius melpomene* and *Papilio polytes*), female butterflies perceive oviposition stimulant by the chemosensilla located on the ventral side of their foreleg tarsus and further determine the suitable feeding plant for larvae [10–12]. In other lepidopteran species (such as *Mnesampela privata*, *Helicoverpa armigera* and *Heliothis virescens*) (Fig 1), tarsal chemosensilla of the prothoracic legs are sensitive to some salts, sugars and amino acids, which indicates a role in the assessment of food materials [13–15]. Legs of *Drosphila*, functioning as gustatory organ and being responsible for tastant recognition, contain several taste sensilla and make the initial contact with potential food resources [16].

Gustatory perception enables insects to efficiently detect the non-volatile chemosensory information, guiding the feeding and oviposition behaviors of insects. Gustatory stimuli are recognized by gustatory receptors (GRs), which share a common 7-transmembrane protein and plus C-terminal domain with olfactory receptors (ORs) but are more diverse [17–19]. According to the ligand profiles, GRs are classified into sugar GR genes [20–22], bitter taste receptors [23–25] and carbon dioxide receptors [26, 27]. Insect OBPs (odorant binding protein) are small, hydrophilic proteins, ferrying the hydrophobic semiochemicals across the sensilla lymph to olfactory receptors [28]. In Lepidoptera, two subfamilies of OBPs, general odorant-binding proteins (GOBPs) and pheromone binding proteins (PBPs), are responsible for recognizing and transporting host odorants and pheromones, respectively [29, 30]. Although OBPs were identified originally in olfactory system, some OBPs appear in gustatory sensilla [31–34]. Recent studies have reported an unexpected role of OBP involved in gustatory perception. In *Drosphila*, *OBP49a* is indispensable for the suppression of sweet taste by bitter chemicals, and the loss of *OBP49a* will impair the inhibition [35]; two OBPs encoded by *OBP57e* and *OBP57d* are not only involved in taste perception but could also change the behavioral response to the host odors, and the mutation in these OBPs could shift the host preference [36]. CSPs (chemosensory protein) are also small soluble proteins enriched in the sensilla lymph [37], unlike OBPs which are considered antennae-specific, CSPs are much smaller (10–15 KDa) but widely expressed in many tissues, including antennae [38, 39], proboscises [40, 41], legs [42], wings [43], etc. In general, CSPs are believed to enhance the solubility of semiochemicals and deliver them to chemosensory receptors [44–47], however, the function remains unclear. SNMPs (sensory neuron membrane proteins), transmembrane domain-containing proteins, are expressed in pheromone-sensitive hair and proposed to participate in pheromone and general odorant reception [48–50].

The tea geometrid, *Ectropis obliqua* Prout (Lepidoptera: Geometridae) is one devastating chewing pest throughout the tea plantations in China. *E*. *obliqua* larvae, voracious worms that feed exclusively on tea leaves and tender buds, produce 6–7 generations throughout the growing season of tea, and eat the entire leaves of tea plants in several infestations, causing severe yield loss and deterioration in commercial tea quality [51]. As one member of Lepidoptera, the ability to locate suitable host plants is fundamental to the survival of their offspring, because the small larvae cannot easily forage for alternate hosts [52]. Field observation of the egg-laying behavior in *E*. *obliqua* reveals the female adults actively forage for the conspecific larvae-infested plants and preferentially oviposit on the splits of branches or the cracks between leaves and branches, during which the phytochemical compounds in leaves are the dominant attraction for oviposition [53, 54]. Thus, a deep insight into the molecular mechanisms of gustatory perception will largely contribute to the integrated management of *E*. *obliqua*.

Previous studies of gustatory system mainly focused on the electrophysiological recording of gustatory sensilla and behavioral response, and were confined to the limited butterflies and model species, such as *Bombyx mori* and *Drosophila melanogaster*, whose genomes were available. However, few studies were focused on agricultural pests and the underlying genetic mechanisms were poorly understood, primarily because of the difficulty in detecting the genes involved in taste sensation. Here we performed Illumina HiSeq 2500 sequencing of the transcriptome of adult male and female legs of this devastating economic pest, and reported the identification of 24 candidate OBPs (including 4 PBPs), 21 CSPs, 2 SNMP, 3GRs and 4 ORs genes. The transcripts analysis of RPKM metric was performed to highlight the most abundant genes and outline the comparative gene expression between samples. Through RT-PCR and real-time quantitative-PCR, we analyzed expression profiles of the chemosensory genes and their putative functions in chemoreception.

## Materials and Methods

### Insect material and RNA preparation

*E*. *obliqua* colony used in this study was originally collected from the experimental tea plantation of the Tea Research Institute of the Chinese Academy of Agricultural Sciences (Hangzhou, Zhejiang, China). Newly hatched larvae were transferred onto fresh tea shoots in enclosed nylon mesh cages (70×70×70 cm) and kept in a climate-controlled room at 25±1°C and 70±5% relative humidity under a photoperiod of 14:10 (light: dark). After pupation, male and female pupae were separated based on the body size and morphological characters [55], and kept in darkness until eclosion. After emergence, adult moths were given a 10% honey solution. For transcriptome sequencing, legs of unmated male and female individuals were collected 2–3 days after eclosion. For qPCR and RT-PCR, adult tissues were collected and divided into female antennae, male antennae, male legs, female legs, heads (without antennae), thoraxes, abdomens and wings. All tissues were immediately snap-frozen in liquid nitrogen, and stored at –80°C until extraction. Total RNA was extracted using Trizol reagent (Invitrogen, Shanghai, China). The integrity of RNA samples was detected by gel electrophoresis, and a NanoDrop 2000 spectrophotometer (NanoDrop, Wilmington, DE, USA) was used to determine RNA quantity.

### Scanning electron microscopy (SEM)

Prothoracic tarsi of female moths were cut using a scalpel. The samples were first soaked in 70% ethanol for 3 h and then descaled in an ultrasonic bath for 10 s. After gradient elution in an ethanol series (80%, 90%, 95% and 100%), the samples were dried at 25°C overnight. The samples were mounted on stainless steel holders and coated with gold-palladium. Photomicrographs were viewed with a Quauta 200 FEG Environmental scanning electron microscopy (ESEM) (Feicompany, USA).

### cDNA library construction and Illumina sequencing

cDNA library was constructed using 5 μg total RNA extracted from approximately 100 male or female legs. Oligo (dT) linked beads were used to isolate the mRNA from the total RNA (Illumina; San Diego, CA, USA). The isolated mRNA strands were digested into short fragments with Fragmentation Buffer. The fragmented mRNAs were used as templates to construct the cDNA libraries using a Truseq RNA Sample prep Kit (Illumina, San Diego, USA) following the manufacturer's instruction. Briefly, random hexamer-primers were used for first-strand cDNA, followed by second-strand cDNA synthesis using DNA Polymerase I and RNase H (Invitrogen, Carlsbad, CA). After end-repairing and ligation of adaptors, the products were amplified by PCR and purified with QIAquick PCR purification kit (Qiagen, Hilden, Germany) to construct a cDNA library. Then the two libraries created from the legs of male and female *E*. *obliqua* were sequenced on the Illumina HiSeq 2500 platform at Shanghai Majorbio Bio-pharm Technology Co., Ltd, generating 100 bp paired-end raw reads.

### Transcriptome assembly and functional annotation

Clean-read datasets were obtained from the raw reads through the following procedures: first, remove the low quality reads (the bases with sequencing error rates more than 1% are over half in the read) and adaptor sequences; second, filter out the sequences with N (uncertain bases) exceeding 10%. These treatments were performed through SeqPrep (https://github.com/jstjohn/SeqPrep) and Sickle (https://github.com/najoshi/sickl). The Q20, Q30, GC-content and sequence duplication level of the clean data were summarized simultaneously. Transcriptome assembly was performed through Trinity (trinityrnaseq-r2013-02-25). The trinity outputs made up two classes of unigenes: the consensus cluster sequences and singletons. To acquire the functional annotation, transcripts larger than 150 bp were submitted to NCBI BlastX homology search against a pooled database of non-redundant (nr) and SwissProt protein sequences with an E-value ≤1.0E–5. We further imported the blast results into Blast2GO pipeline for Gene Ontology (GO) annotation. Then, the open reading frame (ORF) of genes was predicted by ORF finder (http://www.ncbi.nlm.nih.gov/gorf/gorf.html), and the SignalP 4.1 server (http://www.cbs.dtu.dk/services/SignalP/) was used to predict the signal peptide sites in the protein sequences. In order to explore the putative chemosensory receptors, the available GRs and ORs sequences from *B*. *mori*, *D*. *melanogaster* and *Tribolium castaneum* were submitted as queries to run local homology search against the assembled transcripts using the BioEdit Sequence Alignment Editor 7.1.3.0, in avoidance of inaccurate gene annotation.

### RACE–PCR and sequence verification

The retrieved unigenes did not always represent full-length transcripts and some contained only part. To confirm the reliability of output sequences and for better resolution of phylogenetic analysis, partial sequences of candidate chemosensory genes were extended using RACE-PCR, and subsequently followed by full-length assembly and cloning. Total RNA extracted from adult antennae or legs was used to synthesize 5’ and 3’ RACE cDNA templates through SMART RACE cDNA Amplification kit (Clontech). Primers were designed manually and listed in S1 Table. The RACE-PCR was operated in the means of touchdown following the manual of Advantage 2 PCR kit (Clontech, CA, USA). The PCR products were subcloned into pGEM-T (promega) and the inserts were sequenced using M13 primers. Afterwards, the 3’ and 5’ end sequences were aligned by BlastX against the GenBank to validate its correctness, and were sequence-matched to obtain the full length. Open reading frames (ORFs) of genes were predicted by ORF finder (http://www.ncbi.nlm.nih.gov/gorf/gorf.html). Then gene specific primers were designed using the Primer 5.0 software, and ORF sequences were amplified and verified by sequencing as mentioned before.

### Phylogenetic analysis

Multiple alignments of the complete OBPs and CSPs amino acid sequences were performed by ClustalX 2.0 and further edited by GeneDoc 2.7. The phylogenetic trees were constructed by MEGA6.0 using the Neighbor-joining method with a p-distance model and a pairwise deletion of gaps. Bootstrap support was assessed by a boot strap procedure based on 1000 replicates. The data sets of OBPs and CSPs sequences which were chosen from other Lepidoptera species were listed in S3 and S4 Tables separately.

### Comparative analysis of transcript abundance

To compare the differential expression of chemosensory genes between samples, the transcript expression abundances were calculated according to the metric RPKM (Reads per Kilobase per million mapped Reads) method, based on the formula: RPKM (A) = (10^6^×C×10^3^)/(N×L), where RPKM represents the expression of target gene A, C is the number of reads that are uniquely mapped to gene A, N is the whole number of reads that are uniquely aligned to all transcripts and L is the number of bases in gene A. RPKM metric is capable of eliminating the discrimination in gene lengths and sequencing discrepancies, which makes it possible to compare gene expression between samples [56]. Differentially expressed genes (DEGs) were identified by EdgeR (http://www.bioconductor.org/packages/2.12/bioc/html/edgeR.html) according to statistically significant differences with the threshold of false discovery rates (PDR)<0.05 and |log_2_Ratio≥1| (refer to Benjamini (2001) for details) in the manner of male transcriptome vs. female transcriptome. Subsequently, all DEGs were further annotated by GO and KEGG pathway enrichment analyses.

### qRT-PCR analysis and RT-PCR confirmation

The tissue expression profiles of chemosensory genes in different tissues (male antennae, female antennae, male legs, female legs, heads without antennae, thoraxes, abdomens and wings) were measured by real-time qRT-PCR. After the digestion of residual genomic DNA from total RNA with DNase I (Promega), cDNAs were synthesized using 1 μg total RNA from various tissues by the Fast Quant RT kit (TIANGEN, Beijing, China) following the instruction manual. qRT-PCR was conducted on an Bio-Rad CFX96 Touch Real-Time PCR Detection System. Specific primer pairs were designed by the Primer3 web program (http://primer3.ut.ee/) and listed in S2 Table. The reference gene β-actin (GenBank accession number KT860051) was used for normalization. To make sure that the amplification efficiencies of target genes and reference gene are approximately equal, the efficiency of each primer pair was analyzed by constructing a standard curve with three-fold cDNA dilution series. The qRT-PCR reaction contained 10 μl SuperReal PreMix Plus (TianGen, Beijing, China), 0.6 μl primer on each (10 μM), 2 μl sample cDNA (200 ng) and sterile H_2_O up to 20 μl. The qPCR procedure was 95°C for 15 min, followed by 40 cycles of 95°C for 10 s and 60°C for 30 s, melt curves stages at 95°C for 15 s, 60°C for 1 min, and 95°C for 15 s. A blank without template cDNA was included in each experiment serving as a negative control. To check reproducibility, each reaction included three biological replicates and was performed in triplicate (technical replicates). Relative transcript level in each tissue was calculated using the comparative 2^–ΔΔCT^ method [57]. Data were first normalized to the endogenous β-actin levels from the same tissue, then the lowest-expression tissue was selected as the calibrator, and the relative expression level among different tissues was assessed by comparing the expression level of each target gene in other parts to that in the lowest one.

RT-PCR was implemented to verify the expression profiles of chemosensory genes. Specific primers were designed by Beacon Designer 7.7 (Premier Biosoft, Palo Alto, CA, USA) and listed in S2 Table. Each PCR reaction contained 200 ng cDNA template, performed by Taq Master Mix (CWBIO, Beijing, China) under general 3-step amplification by 34–36 cycles of 94°C for 20s, 58°C for 30s, 72°C for 40s. PCR products were checked by electrophoresis and further confirmed by sequencing. The β-actin gene served as an endogenous control. Each amplification was performed three times with different biological samples.

### Statistical analysis

Data of relative expression levels in various tissue were subjected to one-way analysis of variance (ANOVA), followed by a least significant difference test (LSD) for mean comparison. Differences were considered significant at p<0.05. Data were analyzed using SAS 9.20 (SAS Institute, Cary, North Carolina, USA).

## Results

### Transcriptome overview

The transcriptome reads data were generated on an Illumina HiSeq 2500 platform using the paired end protocol. A total of 61.9 and 48.1 million raw reads were obtained from the *E*. *obliqua* female and male-leg libraries. After filtering the low quality and adaptor sequences, 59729104 and 46908620 clean reads were obtained, respectively, and assembled together into 83311 transcripts with an average length of 1402 bp. Of the clean reads, the Q20 percentage (proportion of sequences with a sequencing error rate less than 1%) for both libraries exceeded 98%. The clean reads obtained in this study are available at the NCBI/SRA database under the accession number SRX1502449. The length distribution of transcripts and unigenes was listed in S1 Fig.

**Fig 2:**
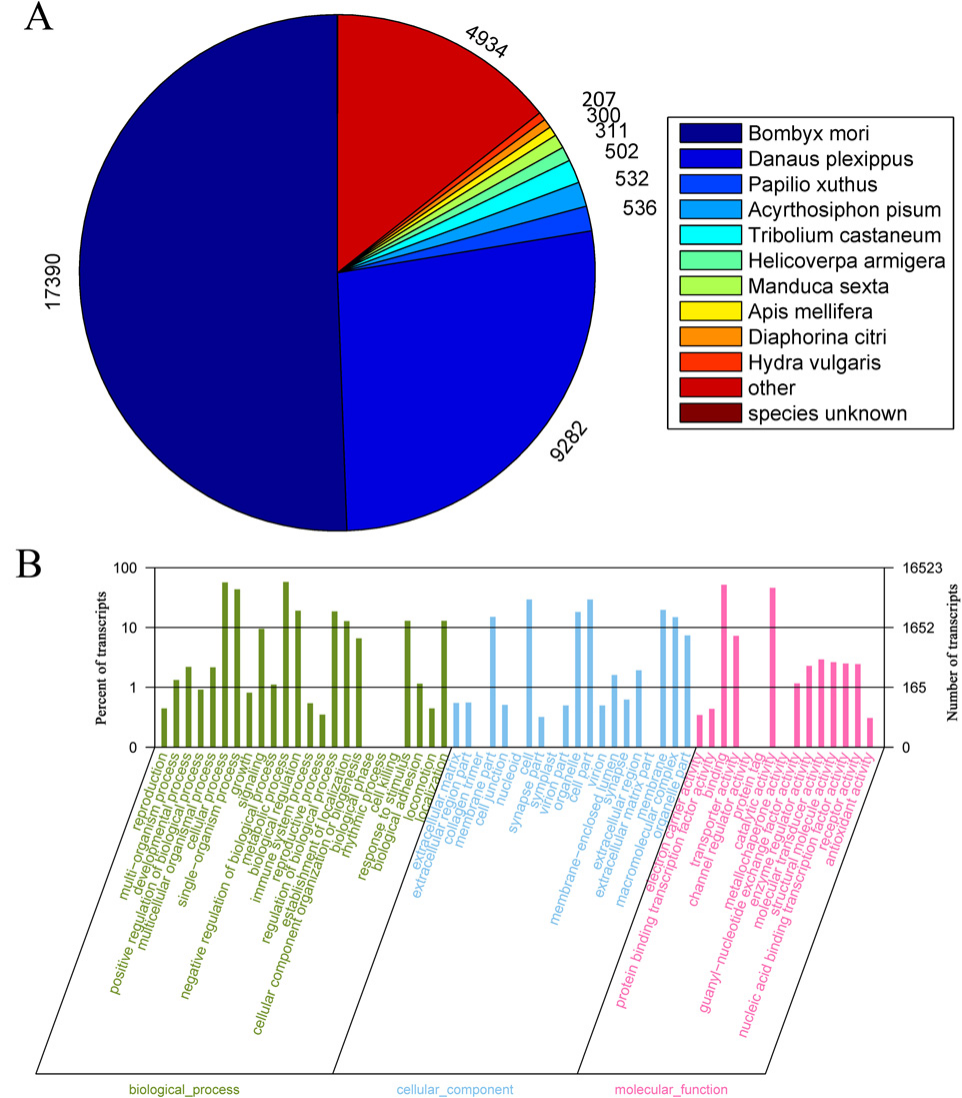
Summary for the annotation of *E*. *obliqua* legs transcripts. (A) Species distribution of the best Blastx hits. (B) Gene Ontology (GO) classifications of legs transcripts annotated at GO level 2 according to the involvement in biological processes, cellular component and molecular function.

The functional annotations of transcripts were performed using the sequence similarity searches against the Nr, SwissProt, KEGG, GO and COG databases with an E-value threshold of 10^−5^. A percentage of 41.3%, 26.1% and 17.9% transcripts hit in Nr, SwissProt, and KEGG database, respectively. Among the annotated transcripts, 17390 (50.6%) of Nr-hit transcripts had a best match to *B*. *mori*, followed by 9282 (27.0%) to *Danaus plexippus* and 4924 (14.3%) to *P*. *Xuthus* (Fig 2A). GO gene functional classification offers a strictly defined concept to depict the properties of genes and their products. Of the total transcripts, only 83311 (19.8%) could be annotated based on sequence homology, and the assembled transcripts were divided into 3 distinct subsets (Fig 2B). In the term of molecular function, the annotations were mostly enriched in binding (8539 transcripts accounting for 51.7% of the annotated) and catalytic activity (7600 transcripts accounting for 46.0%). In biological process category, metabolic and cellular processes occurred most frequently; while the cell, cell part and organelle were most abundant in the category of cellular component.

### Identification of putative chemosensory genes

**Fig 3:**
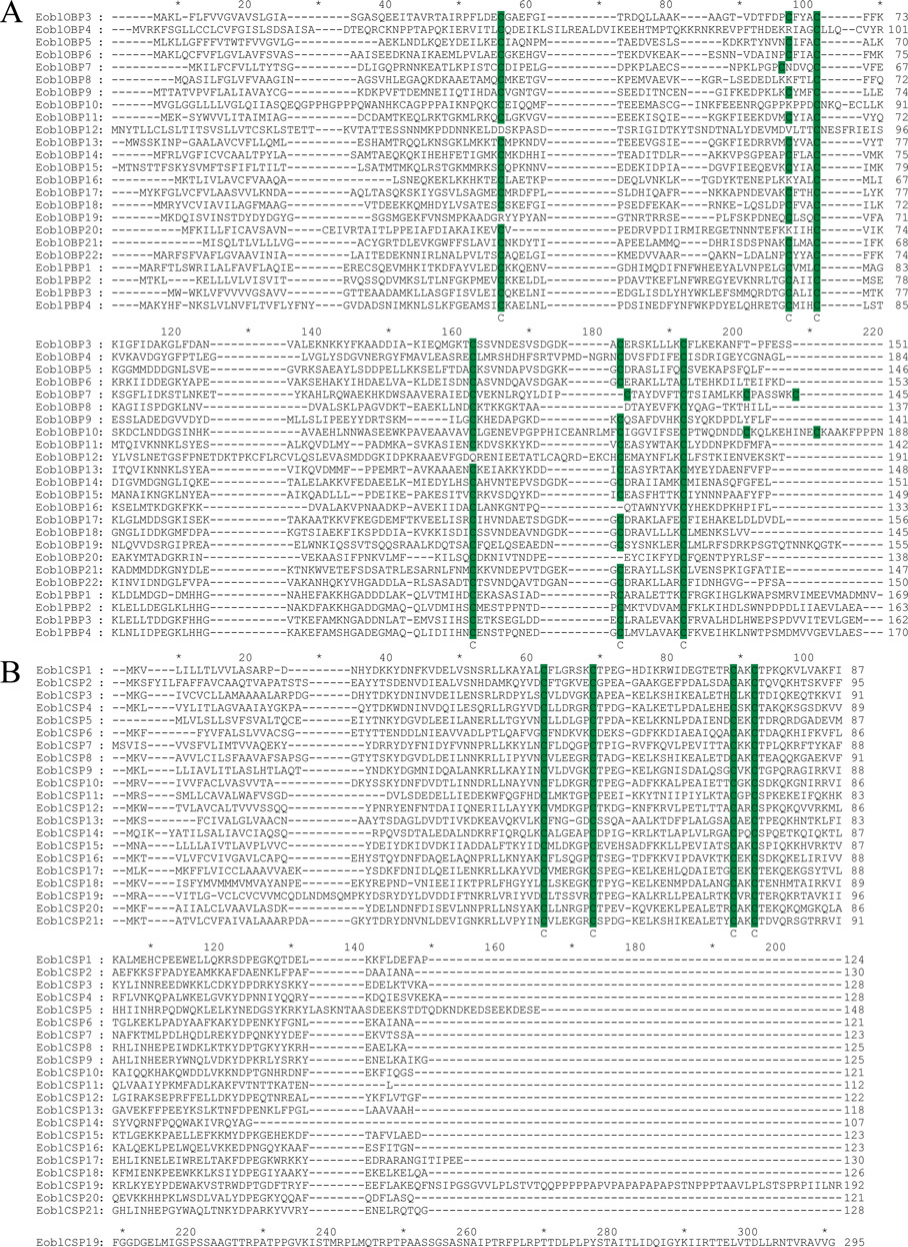
Alignment of amino acid sequences of candidate OBPs and CSPs from *E*. *obliqua*. (A) Amino acid alignment of the candidate OBPs. (B) Amino acid alignment of the candidate CSPs. Green boxes show the conserved cysteine residues. Accession numbers of the *E*. *obliqua* OBPs and CSPs are listed in Table 1.

**Fig 4:**
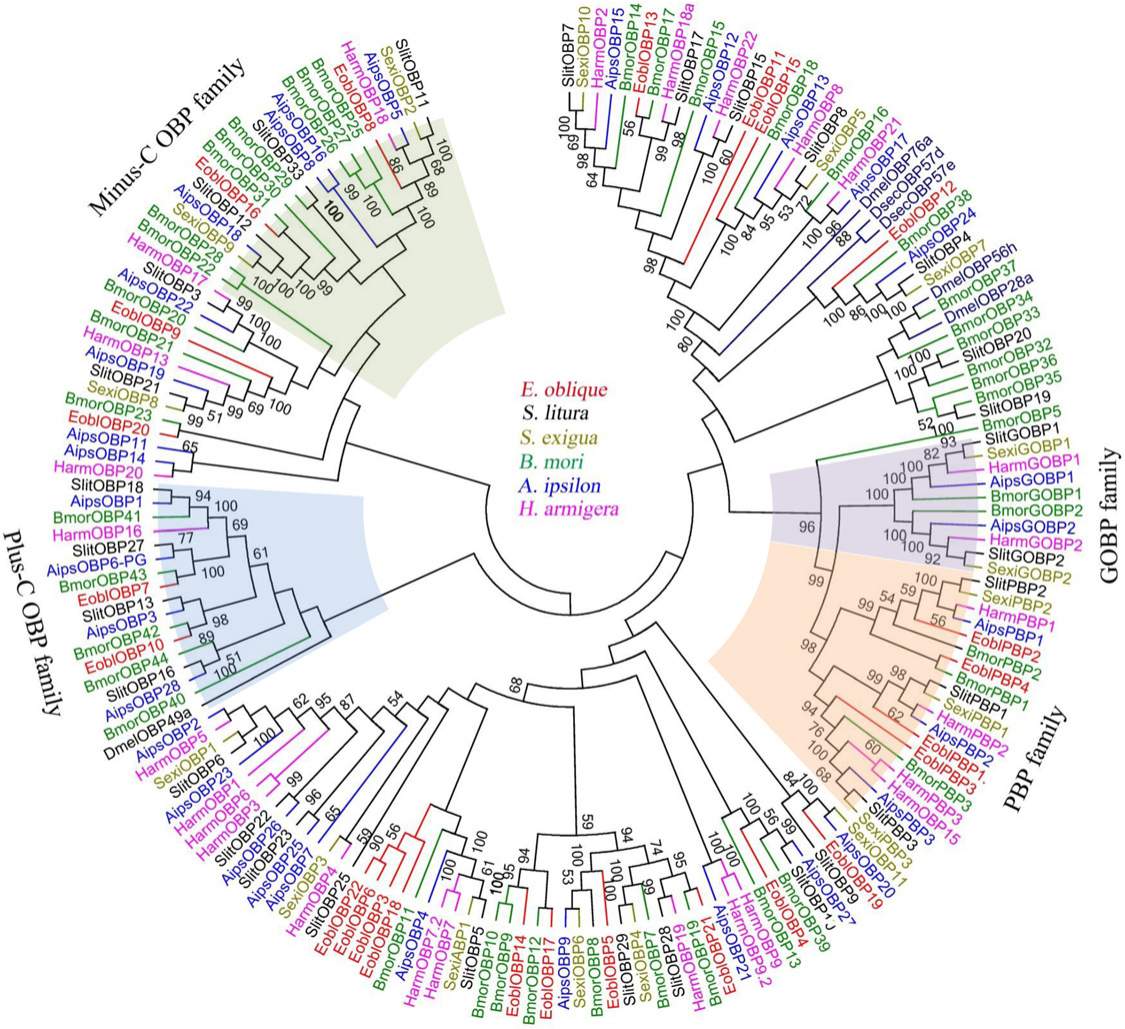
Neighbor-joining tree of candidate OBP proteins from Lepidoptera species. The protein names and sequences of the 178 OBPs used in this analysis are listed in S3 Table. Eobl, *E*. *obliqua*; Slit, *S*. *litura*; Sexi, *S*. *exigua*; Bmor, *B*. *mori*; Aips, *A*. *ipsilon*; Harm, *H*. *armigera*.

Sequence annotation facilitated the identification of candidate chemosensory genes, generating a total of 24 OBPs (including 4 PBPs), 21 CSPs, 2 SNMP, 3GRs and 4 ORs genes (Table 1). Sequence predication revealed that 20 OBPs included the full-length open reading frame (ORF) with a predicted signal peptide, and the five incomplete OBPs were extended in either 5 or 3 directions by RACE-PCR. The ORF sequences of 24 EoblOBPs were verified by cloning and sequencing and further submitted to GenBank (Table 1). After acquiring the full length sequence, the 24 putative EoblOBPs shared a relative high similarity (34%-92%) to the known lepidopteran OBPs, among which *EoblOBP20*, *EoblPBP1* and *EoblPBP2* were matched to the orthologous gene from the Geometridae relative *Ascotis selenaria*. Based on the number and location of the conserved cysteines, 24 EoblOBPs can be divided into three subsets. *EoblOBP8*, *EoblOBP16* and *EoblOBP20* are classified as the Minus-C OBP family, which lack the conserved cysteines C2 and C5 (Fig 3A); while *EoblOBP7* and *EoblOBP10* belong to the Plus-C OBP family, which have extra 2 cysteines located behind the conserved C6. Moreover, the conserved C2 and C3 of these two Plus-C OBPs are separated by 4 amino acid residues rather than typical 3 in classic OBP, and the conserved C5 and C6 of *EoblOBP7* are separated by 7 amino acid residues rather than usual 8 as in the Plus-C OBP (Fig 3A). The 19 EoblOBPs left are classic OBPs with the typical character of six conserved cysteines. The 24 EoblOBPs along with 153 OBPs from 7 other species (including *B*. *mori*, *H*. *armigera*, *Agrotis ipsilon*, *Spodoptera exigua*, *Spodoptera litura*, etc.) were chosen to construct a phylogenetic tree based on the amino acid sequences. The tree could be classified into several distinct branches: the PBP family, the GOBP family, the Plus-C OBP family and the Minus-C OBP family (Fig 4). On the whole, the identified EoblOBPs were clustered with different orthologous sequences in other species, except that *EoblOBP3*, *EoblOBP6*, *EoblOBP18* and *EoblOBP22* made up a homologous cluster. Besides, nine EoblOBPs were coupled with corresponding homologous OBPs from *B*. *mori* in one branch.

**Table 1:** Chemosensory genes in *E*. *obliqua* male- and female- legs transcriptome.

GeneName	Accession No.	Length(bp)	CompleteORF	ORF(aa)	Best Blast P Match	E value	Iden-tity	RPKM value		log2FC	FDR	up-down- regulation	signifi -cant
								Female	Male				
**Odorant Binding Protein (OBP)**													
OBP3	KT327208	618	Yes	151	gb|EHJ67766.1|antennal binding protein [Danaus plexippus]	1e-35	49%	16.49	6.07	1.5832	8.20E-07	up	yes
OBP4	KT327209	990	Yes	184	gb|AKT26501.1|odorant binding protein 24 [Spodoptera exigua]	2e-123	92%	23.31	43.95	-0.8929	3E-04	down	no
OBP5	KT327210	733	Yes	146	gb|AII00976.1|odorant binding protein [Dendrolimus houi]	2e-40	56%	92.57	42.3	1.2555	2.55E-07	up	yes
OBP6	KT327211	660	Yes	153	gb|AKT26503.1|odorant binding protein 26 [Spodoptera exigua]	4e-36	43%	4452.85	7846.6	-0.3940	0.16514	down	no
OBP7	KT327212	591	Yes	145	gb|AGK24580.1|odorant-binding protein 4 [Chilo suppressalis]	2e-67	68%	7.68	6.52	0.4119	0.44031	up	no
OBP8	KT327213	567	Yes	137	gb|AII01007.1|odorant binding protein [Dendrolimus kikuchii]	5e-48	63%	2325.08	3066.73	-0.2268	0.49493	down	no
OBP9	KT327214	590	Yes	141	gb|AFD34173.1|odorant binding protein 5 [Argyresthia conjugella]	2e-70	67%	67.24	183.35	-1.2827	7.15E-08	down	yes
OBP10	KT327215	969	Yes	188	gb|AII01009.1|odorant binding protein [Dendrolimus kikuchii]	5e-25	36%	1987.8	1775.41	0.2521	0.43405	up	no
OBP11	KT327216	560	Yes	142	gb|AGM38605.1|odorant binding protein [Chilo suppressalis]	2e-61	64%	83.49	12.22	2.9454	7.87E-29	up	yes
OBP12	KT327217	693	Yes	191	gb|CAS90131.1|odorant-binding protein 7 [Bombyx mori]	3e-32	53%	4.52	2.91	0.7661	0.14810	up	no
OBP13	KT327218	633	Yes	148	gb|AAL60415.1|antennal binding protein 4 [Manduca sexta]	8e-75	78%	15.61	1.57	3.4075	4.52E-17	up	yes
OBP14	KT327219	669	Yes	151	gb|AGP03457.1|SexiOBP11 [Spodoptera exigua]	8e-67	66%	6.84	1.47	2.1597	4.19E-06	up	yes
OBP15	KT327220	692	Yes	149	gb|AEB54589.1|OBP8 [Helicoverpa armiger]	2e-72	79%	15.02	1	4.1939	5.36E-20	up	yes
OBP16	KT327221	1068	Yes	133	gb|AAA85090.1|sericotropin [Galleria mellonella]	5e-82	91%	172.91	167.91	0.1214	0.77672	up	no
OBP17	KT327222	609	Yes	156	gb|AGS36748.1|OBP6 [Sesamia inferens]	1e-32	53%	89.7	38.11	1.2966	3.33E-08	up	yes
OBP18	KT327223	1065	Yes	145	gb|AII00968.1|odorant binding protein [Dendrolimus houi]	1e-27	40%	30.79	31.06	0.0671	0.93038	up	no
OBP19	KT327224	1090	Yes	155	gb|AKI87962.1|odorant binding protein 1 [Spodoptera litura]	9e-63	71%	3.55	1.29	0.9815	0.02123	up	no
OBP20	KT327225	672	Yes	138	gb|KOB71255.1|Odorant binding protein [Operophtera brumata]	1e-15	34%	0	0.52	noTest	noTest	noTest	noTest
OBP21	KT762010	597	Yes	147	gb|AII00969.1|odorant binding protein [Dendrolimus houi]	1e-47	48%	8.14	3.5	1.3898	0.00131	up	yes
OBP22	KT762011	592	Yes	150	gb|AKT26503.1|odorant binding protein 26 [Spodoptera exigua]	4e-25	36%	0.83	0	noTest	noTest	noTest	noTest
PBP1	KT282990	856	Yes	169	dbj|BAF63878.1|pheromone binding protein [Ascotis selenaria]	7e-91	78%	13.21	9.5	0.5794	0.09273	up	no
PBP2	KT282991	1178	Yes	164	dbj|BAF64703.1|pheromone binding protein 2 [Ascotis selenaria]	2e-69	76%	22.98	27.6	-0.1930	0.62504	down	no
PBP3	KT282992	845	Yes	162	gb|AIS72934.1|pheromone binding protein 3 [Spodoptera litura]	4e-55	57%	59.18	62.18	0.03240	0.99049	up	no
PBP4	KT282993	1115	Yes	170	gb|AAF06142.1|pheromone binding protein [Synanthedon exitiosa]	2e-69	63%	15.14	12.09	0.4359	0.20968	up	no
**Chemosensory Protein (CSP)**													
CSP1	KT282970	526	Yes	124	gb|AKT26488.1|chemosensory protein 12 [Spodoptera exigua]	5e-45	61%	1.6	1.13	0.7293	0.48061	up	no
CSP2	KT762012	592	Yes	131	gb|AHC05672.1|chemosensory protein [Chilo suppressalis]	2e-24	42%	0.17	1.05	-2.2701	0.20298	down	no
CSP3	KT282971	541	Yes	128	gb|ABM67689.1|chemosensory protein 2 [Spodoptera exigua]	2e-46	61%	23512.11	12405.51	1.1052	1.78E-06	up	yes
CSP4	KT282972	861	Yes	128	gb|AGR39576.1|chemosensory protein 6[Agrotis ipsilon]	4e-64	76%	12169.58	9274.41	0.4949	0.06465	up	no
CSP5	KT282973	543	Yes	148	gb|AGY49270.1|chemosensory protein [Sesamia inferens]	1e-52	78%	7.67	4.46	0.8364	0.01005	up	no
CSP6	KT282974	551	Yes	121	gb|ADO95154.1|chemosensory protein [Antheraea yamamai]	1e-40	58%	2771.92	1722.79	0.8649	3E-04	up	no
CSP7	KT282975	505	Yes	123	gb|KOB75937.1|Chemosensory protein 15 [Operophtera brumata]	6e-56	69%	11.77	7.23	0.8994	0.02407	up	no
CSP8	KT282976	725	Yes	125	gb|ABM67688.1|chemosensory protein 1 [Spodoptera exigua]	5e-51	60%	1013.44	345.55	1.5931	1.36E-12	up	yes
CSP9	KT282977	606	Yes	125	gb|AII01011.1|chemosensory protein [Dendrolimus houi]	2e-45	59%	0.15	7.66	-5.3451	1.02E-15	down	yes
CSP10	KT282978	1184	Yes	121	gb|KOB75936.1|Chemosensory protein [Operophtera brumata]	8e-67	79%	36.34	45.92	-0.2684	0.42525	down	no
CSP11	KT282979	563	Yes	112	gb|AJP61962.1|chemosensory protein [Phenacoccus solenopsis]	4e-07	41%	0.66	87.3	-6.9890	2.69E-103	down	yes
CSP12	KT282980	822	Yes	122	gb|EHJ73333.1|chemosensory protein 13 [Danaus plexippus]	4e-64	85%	3.77	7.61	-0.8258	0.02528	down	no
CSP13	KT282981	590	Yes	118	ref|NP_001037068.1|chemosensory protein 7 [Bombyx mori]	1e-16	37%	12.94	3.62	1.9459	2.22E-09	up	yes
CSP14	KT282982	734	Yes	107	gb|AIW65101.1|chemosensory protein [Helicoverpa armigera]	3e-61	85%	17.32	17.88	-0.0497	0.97152	down	no
CSP15	KT282983	676	Yes	123	gb|AKT26491.1|chemosensory protein 16 [Spodoptera exigua]	6e-37	69%	8.36	175.46	-4.9581	8.21E-80	down	yes
CSP16	KT282984	612	Yes	123	ref|NP_001037067.1|chemosensory protein 8 [Bombyx mori]	1e-53	64%	9.3	4.01	1.2743	3.60E-05	up	yes
CSP17	KT282985	1184	Yes	130	gb|AHX37218.1|chemosensory protein 1 [Conogethes punctiferalis]	1e-71	77%	2389.76	2554.64	0.0175	1	up	no
CSP18	KT282986	648	Yes	126	gb|AKI28976.1|chemosensory protein 2 [Bactrocera dorsalis]	7e-32	51%	1640.61	620.15	1.1747	3.23E-07	up	yes
CSP19	KT282987	1803	Yes	295	gb|AIW65104.1|chemosensory protein [Helicoverpa armigera]	2e-108	65%	42.91	63.32	-0.5197	0.05756	down	no
CSP20	KT282988	698	Yes	121	gb|AEX07265.1|CSP2 [Helicoverpa armigera]	1e-52	78%	40399.5	23894.02	0.8912	2E-04	up	no
CSP21	KT282989	789	Yes	128	gb|ABM67688.1|chemosensory protein 1 [Spodoptera exigua]	2e-58	65%	283.4	33.05	3.2140	9.14E-42	up	yes
**Olfactory Receptor (OR)**													
ORco	KT373968	1693	Yes	473	gb|BAG71418.1|olfactory receptor-2 [Diaphania indica]	0.0	85%	0	0.45	noTest	noTest	noTest	noTest
OR1	KT860045	2683	Yes	438	ref|NP_001091791.1|candidate olfactory receptor [Bombyx mori]	4e-78	40%	1.64	0.54	1.6351	1.63511	up	yes
OR2	KT860046	1931	Yes	391	gb|AII01092.1| odorant receptor [Dendrolimus kikuchii]	2e-146	59%	0.46	0.42	noTest	noTest	noTest	noTest
OR3	KT860047	1580	Yes	402	gb|AJF23815.1|olfactory receptor OR59 [Planotortrix octo]	0.0	74%	0.74	0.4	noTest	noTest	noTest	noTest
**Gustatory Receptor (GR)**													
GR1	KT860048	1756	Yes	351	gb|KDR12697.1|gustatory receptor 1 [Zootermopsis nevadensis]	8e-121	62%	1.21	1.38	-0.1306	0.96147	down	no
GR2	KT860049	1676	Yes	466	gb|AGA04648.1|gustatory receptor [Helicoverpa armigera]	0.0	75%	3.81	5.07	-0.4306	0.21840	down	no
GR3	KT860050	1589	3' lost	423	gb|AGK90023.1|gustatory receptor 1 [Helicoverpa assulta]	0.0	72%	0.37	0.32	noTest	noTest	noTest	noTest
**Sensory Neuron Membrane Protein (SNMP)**													
SNMP1	KT282969	1908	Yes	524	gb|AGN52676.1|sensory neuron membrane protein 1 [Spodoptera exigua]	0.0	65%	0	0.4	noTest	noTest	noTest	noTest
SNMP2	KP684219 *	1885	Yes	516	gb|AKN78949.1|sensory neuron membrane protein 2 [Ectropis obliqua]	0.0	99%	92.38	530.35	-2.4820	2.55E-28	down	yes

Log2FC: fold change value after log2 transformation of expression levels in the manner of the female legs transcriptome vs. the male legs transcriptome. Differentially expressed genes were identified according to statistically significant differences with the threshold of false discovery rates (PDR)<0.05 and |log2Ratio≥1|. If no transcript representing the target gene was detected, RPKM value and the following analysis should not be taken into account.

* represents that the gene has been submitted already.

**Fig 5:**
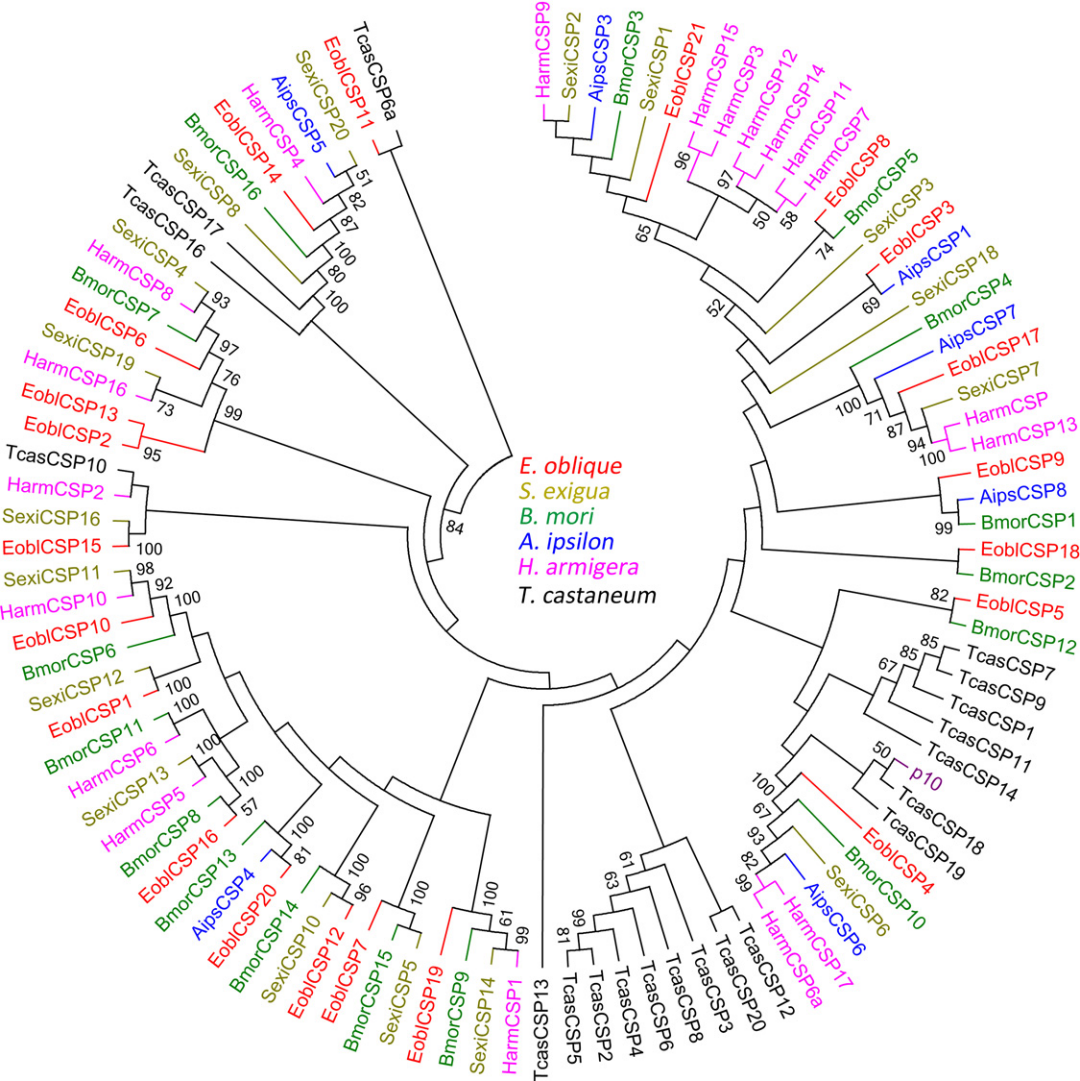
Neighbor-joining tree of candidate CSP proteins from Lepidoptera species. The protein names and sequences of the 101 CSPs that were used in this analysis are listed in S4 Table. Eobl, *E*. *obliqua*; Tcas, *T*. *castaneum*; Sexi, *S*. *exigua*; Bmor, *B*. *mori*; Aips, *A*. *ipsilon*; Harm, *H*. *armigera*. p10 is one CSP reported in the cockroach *Periplaneta Americana* (Kitabayashi et al., 1998).

By homology analysis, we identified 21 transcripts encoding candidate CSPs in *E*. *obliqua*, among which 20 of the 21 EoblCSP genes had intact ORF with a signal peptide and four conserved cysteine residues, which represented the typical character of insect CSPs (Fig 3B). Sequence analysis revealed, relative to EoblOBPs, the 21 EoblCSPs were relatively conserved, the average identify of which was 64.8%. In phylogenetic analysis of CSPs from Lepidoptera species, EoblCSPs were spread across branches where they generally formed separate clusters with others. Only *EoblCSP2* and *EoblCSP13* formed one subgroup (Fig 5).

Two SNMPs were identified from our transcriptome and acquired the full length by RACE-PCR. Both *EoblSNMP1* and *EoblSNMP2* shared more than 60% (65% and 69%) identity with the corresponding SNMPs in *S*. *exigua*. The transcripts encoding chemosensory receptors were initially identified by the keyword search of functional annotation, and further confirmed by the local homology search. Four ORs and three GRs were easily identified with 7 transmembrane domains. From the annotation, *EoblGR2* shared 75.2% identity with *HarmGR9* which had been identified as a sugar receptor [58, 59].

### Expression profiles of chemosensory genes

**Fig 6:**
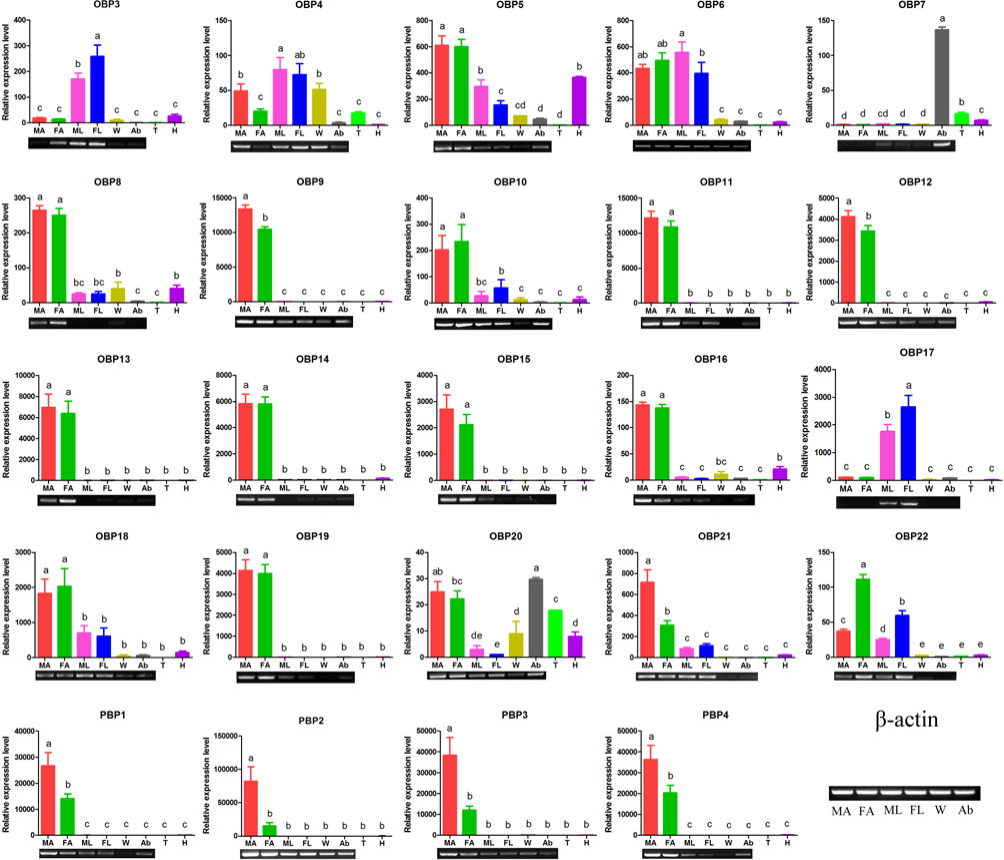
Tissue- and sex- specific expression profiles of *E*. *obliqua* OBP genes by qRT-PCR analysis and RT-PCR confirmation. FA: female antennae, MA: male antennae, FL: female legs, ML: male legs, H: heads, T: thoraxes, A: abdomens, L: legs, W: wings. In qPCR data were first normalized to endogenous β-actin levels from the same tissue, and the lowest-expression tissue was selected as the calibrator. The standard error is represented by the error bar, and the different letters above each bar represent significant differences (p<0.05). EoblOBPs expression of the former six tissues were confirmed by RT-PCR and arranged in the same order as that of qRT-PCR. β-actin was used as an internal reference gene to test the integrity of each cDNA template.

The expression profiles of chemosensory genes (OBPs, CSPs, SNMPs, ORs and GRs) were first examined by qRT-PCR and further confirmed by RT-PCR, illustrating that the majority of EoblOBPs were abundant in antennae (Fig 6). 16 of 24 total EoblOBPs (*OBP8-16*, *OBP18-19*, *OBP21* and *PBP1-4*) were uniquely or primarily expressed in the male and female antennae; while three EoblOBPs (*OBP3-4* and *OBP17*) were highly expressed in legs; *EoblOBP7* were primarily detected in the abdomen. However, the remaining 5 OBPs were distributed in varying tissues, among which *EoblOBP5*, *EoblOBP6* and *EoblOBP22* were primarily enriched in antennae and legs.

**Fig 7:**
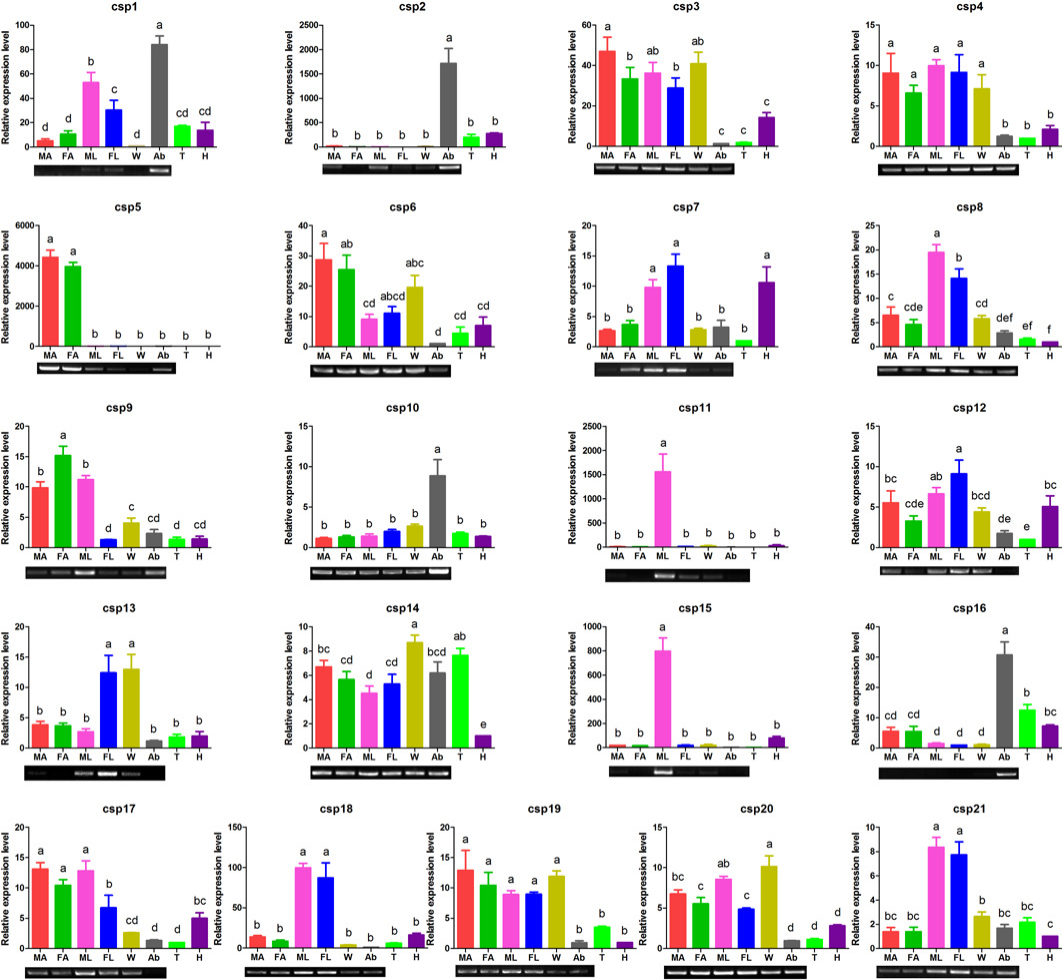
Tissue- and sex- specific expression profiles of *E*. *obliqua* CSPs genesc by qRT-PCR analysis and RT-PCR confirmation. The details were same as mentioned in Fig 6.

The expression pattern of 21 EoblCSPs showed diverse and wide expression (Fig 7). Six EoblCSPs (*CSP7*, *CSP8*, *CSP11*, *CSP15*, *CSP18* and *CSP21*) were dominantly expressed in legs, among which *EoblCSP11* and *EoblCSP15* were highly enriched in male legs. *EoblCSP2*, *EoblCSP10* and *EoblCSP16* were mostly distributed in abdomen, while *EoblCSP6* were uniquely expressed in antennae. The other EoblCSPs were ubiquitous in most tissues. In addition, *EoblCSP1*, *EoblCSP3*, *EoblCSP4*, *EoblCSP9*, *EoblCSP12*, *EoblCSP13*, *EoblCSP14*, *EoblCSP17*, *EoblCSP19* and *EoblCSP20* were abundant in legs at a relatively high level.

**Fig 8:**
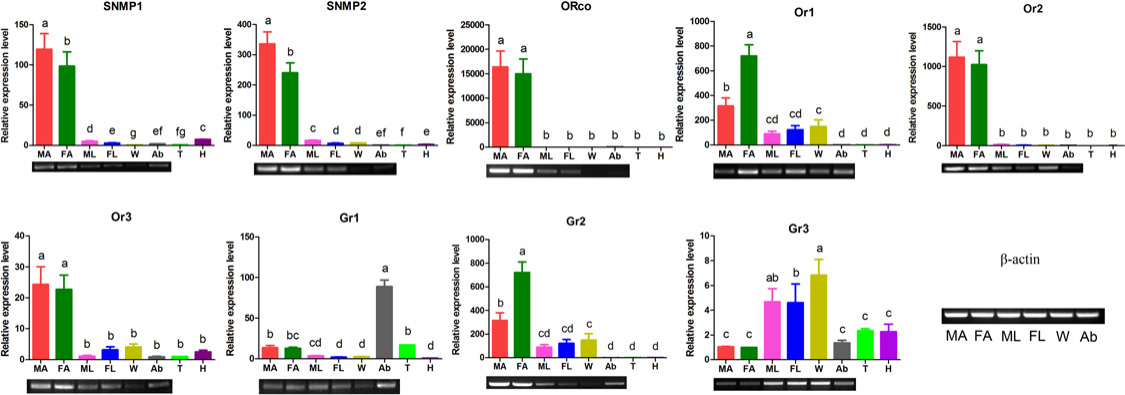
Tissue- and sex- specific expression profiles of *E*. *obliqua* chemoreceptor genes by qRT-PCR analysis and RT-PCR confirmation. The details were same as mentioned in Fig 6.

We also characterized the expression levels of ORs, GRs and SNMP in different tissues (Fig 8). The results indicated that the *EoblSNMP1* and *EoblSNMP2* were expressed significantly higher in antennae than in other tissues of both sexes. Four ORs were mainly expressed in the moth antennae. In addition, the transcript level of *EoblORco* exceeded 15000 fold changes relative to the lowest expression in thoraxes. Among the three EoblGRs identified, *EoblGR1* were enriched in abdomen and *EoblGR2* had antennae-enriched expression, while *EoblGR3* were detected in both legs and wings.

### Abundant analysis of chemosensory genes

**Fig 9:**
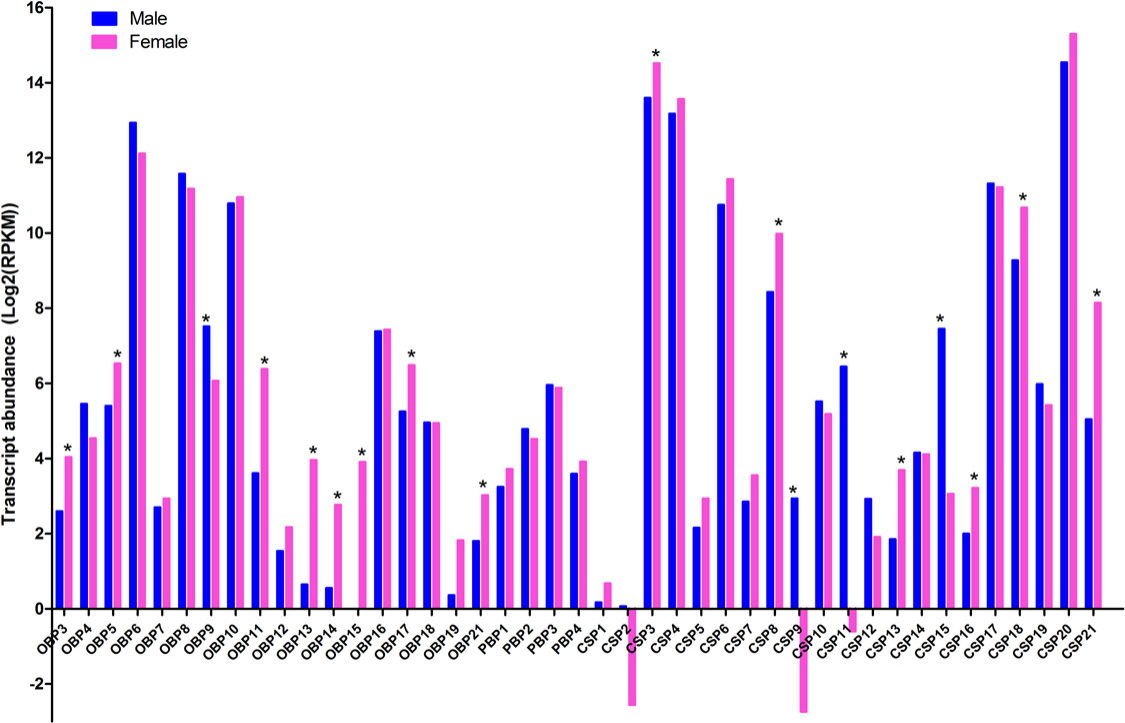
Transcript abundant analysis of chemosensory genes. In single-end RNA-Seq, the transcript expression abundance was calculated with FPKM value. Differentially expressed genes were identified with the threshold of false discovery rates (PDR)<0.05 and |log_2_Ratio≥1|. The significant difference between female and male legs samples was indicated by symbol “*”.

RPKM metric was calculated to evaluate the comparative expression abundance in male and female legs. Of the 24 EoblOBPs, *EoblOBP6* showed the highest expression (7846.6 RPKM in male transcriptome), followed by *EoblOBP8* and *EoblOBP10*. Among the 21 EoblCSPs, *EoblCSP20* was the most abundant (40399.5 RPKM in female transcriptome), followed by *EoblCSP3*, *EoblCSP4*, *EoblCSP17* and *EoblCSP6* (Fig 9). Overall, the levels of expression of EoblOBPs in leg transcriptome were extremely variable, with RPKM values ranging from 1 to 7846; while the EoblCSP expressions were more diverse, from less than 10 to 40399. For chemosensory receptors, both ORs and GRs remained low expression in legs (<10 RPKM), of which *EoblGR2* had the highest expression in both sexes (3.81 RPKM in female and 5.07 RPKM in male). In addition, relative to other transmembrane proteins, *EoblSNMP2* showed an unexpectedly high expression (92.38 RPKM in female and 530.35 RPKM in male).

**Fig 10:**
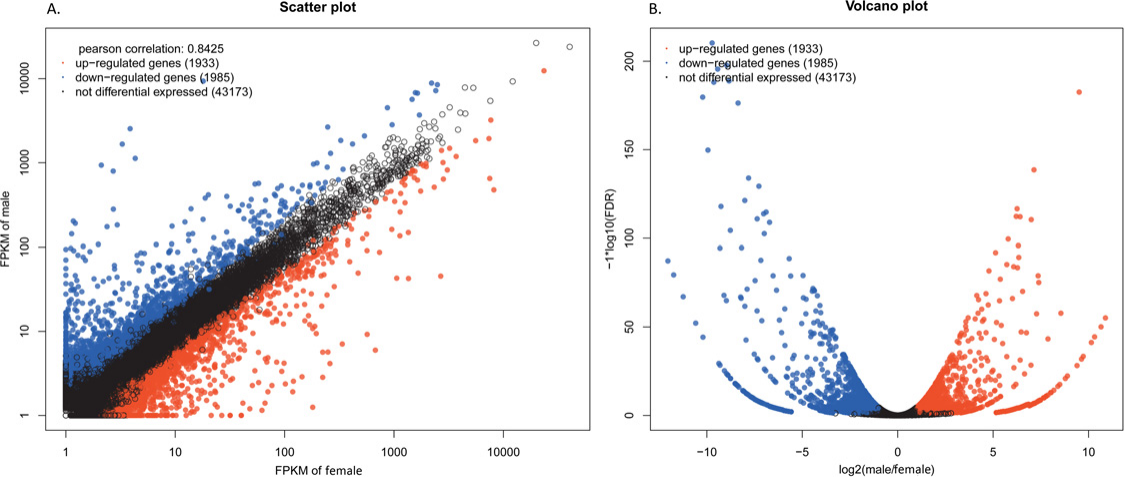
Analysis of differentially expressed genes exhibited in (A) Scatter plot and (B) Volcano plot. Genes are divided into three distinct subsets: blue genes represent the up-regulated ones in the female legs transcriptome vs. the male legs transcriptome, red genes are the down-regulated class compared in the same way, and black part represents the non-differentially expressed transcripts. Differentially expressed genes are identified according to statistically significant differences with the threshold of false discovery rates (PDR)<0.05 and |log2Ratio≥1|.

**Fig 11:**
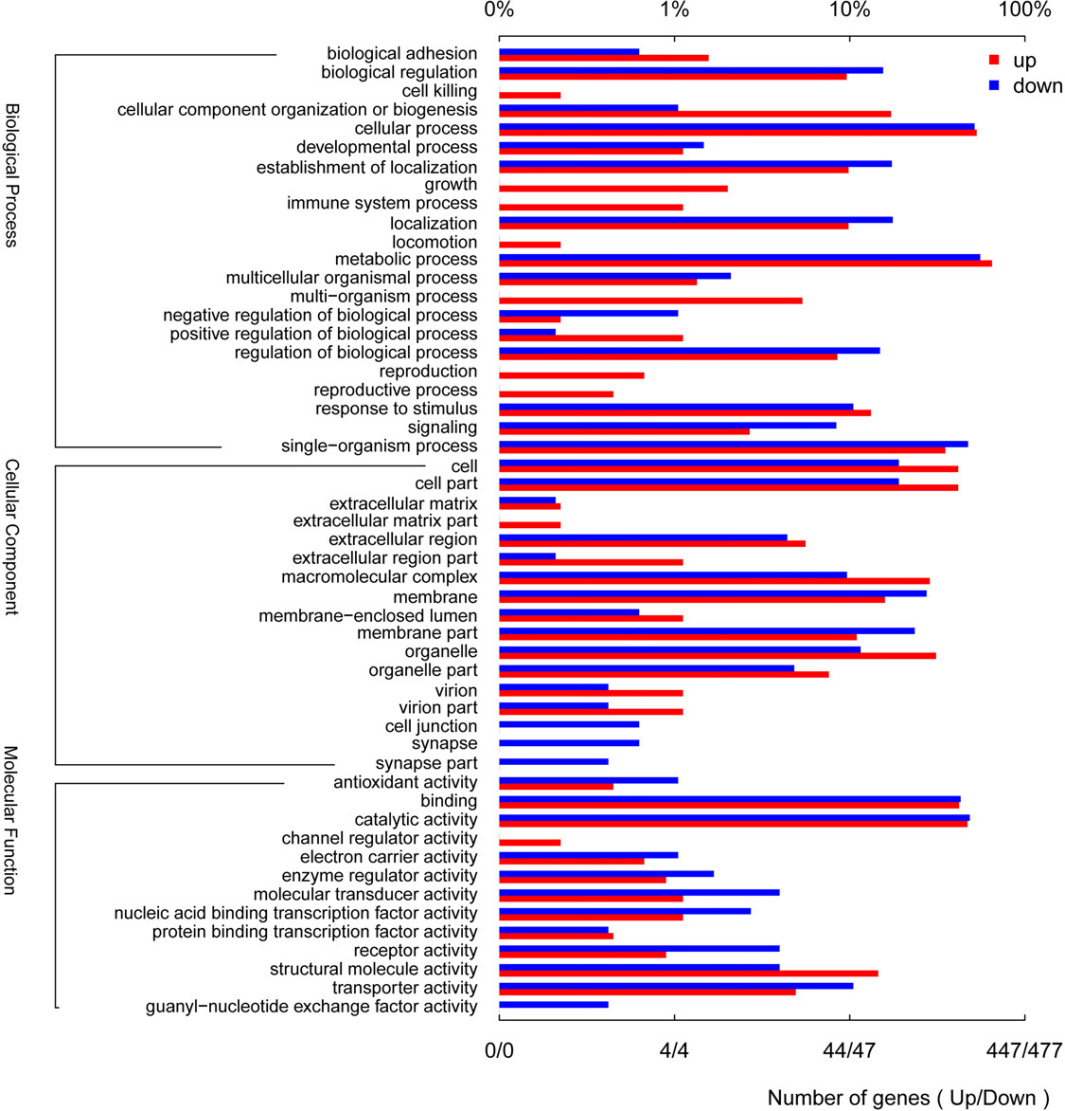
Gene Ontology (GO) enrichment analysis of all the differentially expressed genes. Horizontal axis in the top displays the percentage of significant genes in each column, while axis in the bottom is the number of significant genes. Vertical axis displays the detailed GO annotation corresponding to each functional type. Differentially expressed genes are compared in the manner of the female legs transcriptome vs. the male legs transcriptome.

After summarizing the gene comparative expression between samples, a total of 1933 and 1985 up- and down-regulated unigenes, respectively, showed significantly altered expression (FDR≤0.05 and |log_2_Ratio|≥1), as compared to the male transcriptome. The majority of the unigenes (91.6%), however, were expressed within a two-fold difference (Fig 10). For transporters, 9 EoblOBPs showed sex discrepancy in their levels of legs expression, 8 (*OBP3*, *OBP5*, *OBP11*, *OBP13*, *OBP14*, *OBP15*, *OBP17* and *OBP21*) being over expressed in female and only 1 (*OBP9*) over expressed in male; while 8 EoblCSPs presented sex differences in their expressions, 5 (*CSP3*, *CSP8*, *CSP13*, *CSP16* and *CSP18*) being more expressed in female and only 3 (*CSP9*, *CSP11* and *CSP 15*) up-regulated in male (Fig 9). Unexpectedly, relative to other chemosensory receptors, *EoblSNMP2* showed an abundant expression level and was 4.7-fold greater expressed in male legs. Go classification of the significantly regulated genes was implemented to identify the functional processes involved in sex differences (Fig 11). Overall, these regulated genes were mainly concentrated on cellular process, metabolic process, single-organism process, binding and catalytic activity. Besides, several subcategories were only involved in one-sex-regulation, such as reproduction, growth, multi-organism process and etc.

## Discussion

Host plant selection by herbivorous insects is particularly important for reproduction, involved in searching, landing, contact evaluation, and final determination for oviposition [1]. Generally female adults avoid laying eggs on non-host plants in order to maximize the survival chances of their progenies. Monophagous herbivorous pests, such as *E*. *obliqua*, selectively utilize a limit of host plants, therefore requiring the specialized sensors to explore certain host for oviposition. Olfaction and taste of insects are crucial in detecting and discriminating the chemical compounds from host. In spite that olfaction plays a primary role in perceiving plant volatile odorants from distance, taste is indispensable for non-volatile chemicals recognition after landing on the plant [4]. To ascertain host-plant identity, female butterflies and moths usually contact their forelegs on the leaves of host, which is the first perception of phytochemical compounds when landing on the surface. This initial contact presumably permits insects to taste phytochemical compounds [53]. Consistent with this action, butterflies including *P*. *xuthus*, *H*. *melpomene* and *P*. *polytes* possess groups of trichoid sensilla along with pairs of cuticular spines in female foretarsi, which get involved in the recognition of oviposition stimulants [12, 60, 61]; while 14 gustatory trichoid chemosensilla sensitive to either sugars or amino acids are found in prothoracic legs of moth *H*. *armigera*, *M*. *privata* and *L*. *botrana* [13, 15, 62]. In lepidopteran species, the tarsus is further divided into five tarsomeres, the fifth of which is the most distal part of the tarsus and bears more chemosensory sensilla than the other four tarsomeres. The arrangement of the gustatory sensilla in proximity to prominent tarsal spines is unique and could represent an adaptation which enables them to penetrate the wax layer and contact with metabolites present closer to the leaf surface [13]. Our microscopy of *E*. *obliqua* revealed the distribution of chemosensilla in the ventral side of a female fifth tarsomere (Fig 1), suggesting that the leg tarsi of *E*. *obliqua* were also responsible for taste detection.

Outside the limited butterfly species and model species whose genomes are available, rare studies are focused on gustatory system of other insect species. In fact, the remarkable selectivity and sensitivity of the chemosensory systems depend primarily on the performance of chemosensory neurons, which in turn rely ultimately on odorant receptors, gustatory receptors and selective transporters. So there is a special need to explore the candidate chemosensory genes. From our transcriptome analysis of *E*. *obliqua* legs, 24 OBPs (including 4 PBPs), 21 CSPs, 4 PBPs, 2 SNMP, 3 GRs and 4 ORs genes were identified.

Due to the low expression level of GR [17, 18], only three GR-encoding transcripts were identified from the legs transcriptome. Two of three EoblGRs were highly expressed in abdomen, among which *EoblGR2* shared 75.3% homology with *HarmGR4* that had been identified as a sugar receptor concentrated in larval foregut, female antennae and proleg tarsi of *H*. *armigera* [58, 59]. Thus, we can reasonably assume that *EoblGR2* is also a sugar receptor and could participate in the sugar detection and consumption. The abundance of *EoblGR2* in legs (the highest RPKM value among chemosensory receptors) is of great physiological significance, as most adult lepidopteran insects feed on floral nectar and honeydew, which contributes to female reproductive success [63]. Most ORs in insects are extensively distributed in antennae [64]. The tissue expression profiles of 4 EoblORs demonstrate the obviously antennal-abundance, however, these ORs are also distributed in other organs. The distribution of ORs in non-olfactory tissues suggests that they may participate in other physiological processes besides olfaction. For example, ORco expressed in the testes is involved in mediating activation of spermatozoa in *Anopheles gambiae* [65].

The majority of EoblOBPs (16 in total 24 OBPs) show relatively high expression in antenna, which corresponds to the commonly accepted concept that OBPs function as carriers of hydrophobic ligands to olfactory receptors in antenna [28], however, six EoblOBPs remain highly expressed or relative high in legs. A correlation of some OBPs reported (*OBP49a*, *OBP57e* and *OBP57d*) to host selection [35, 36, 66] and their unexpected distribution in taste organs, such as labellum, wing margins, tarsi, labial palps and etc. [67], raises the possibility that OBPs also participate in taste perception. In fact, non-volatile metabolites in plants are comparable to odorant in the way that they are both small poorly-water-soluble molecules, such as alkaloids and parts of terpenoids [68]. Previous studies have reported the binding of bitter compounds (berberine, denatonium and quinine) to *OBP49a* [35]. Consequently, it is reasonable to conclude that OBPs may act as transporters of hydrophobic compounds to gustatory receptors, which is similar to their performance in olfaction. RPKM metric facilitates the comparative study of expression between samples in mRNA-seq. Our comparative study revealed that 9 EoblOBPs showed sex differences in expression, 8 being up-regulated in female and only 1 being over expressed in male. Previous studies have reported the profound differences in the expression of OBP between sexes [69–71]. Considering that the adult history of male and female moths is quite similar in regard to the aim to fuel their body and the need to mate, the only difference is that females have to identify suitable host for oviposition. This sex difference may have ecological significance as females have to evaluate oviposition sites, so it stands to reason that OBPs with female-biased expression may participate in host selection, and that the female oviposition behavior drives the diversity of OBP expression between sexes.

The phylogenetic analysis reveals most EoblOBPs are clustered with different orthologous sequences from other species, suggesting that the Lepidoptera OBPs have differentiated into several different groups after a long time evolution. However, *EoblOBP3*, *EoblOBP6*, *EoblOBP18* and *EoblOBP22* share a high identity and are clustered in one branch, indicating recent gene duplication events. Besides, *EoblOBP21* shares 37.6% identity and similar expression profile with *HarmOBP10*, which was previously reported to bind one insect repellent 1-dodecene [72].

CSPs are soluble proteins and believed to play a role which is similar to that of OBPs in the perception of odorants [44–47]. The relative expression patterns of 21 EoblCSPs are diverse and widely distributed. Apart from *EoblCSP5* specially expressed in antenna, three CSPs (*EoblCSP2*, *EoblCSP10* and *EoblCSP16*) are primarily present in abdomen, where they might transport semiochemicals in reproductive organs or sex glands, assisting their release into the environment [44, 73, 74]. Fortunately, *HarmCSP6*, sharing 45.2% homology and closely clustered with *EoblCSP16*, was reported to be highly transcribed in pheromone glands and display high binding affinity for pheromone components [75]. In addition, six EoblCSPs are dominantly expressed in legs, besides, 10 EoblCSPs are abundant in legs at the relatively high level. Among them, *EoblCSP21* shared 59.4% identity with *HarmCSP4* which was detected to be exclusively present in proboscis and could help solubilizing terpenoids present in flower nectar [76]; *EoblCSP4* is exceptionally abundant in legs (9274 RPKM in male legs and 12169 RPKM in female legs), sharing 41.7% homology and closely clustered with *Pamep10* which seemed to be involved in limb regeneration [77]. To our surprise, these functions mentioned above are completely unrelated to chemical communication. Actually, the compact structure of CSPs, their soluble nature and flexible polypeptide folding, permit this protein to bind a variety of ligands and therefore could undertake several tasks in the biological process [78].

In summary, a large number of chemosensory genes were identified in *E*. *obliqua*, and tissue distribution profiles were investigated. Several leg-specific or enriched genes were screened, and clustered with functionally validated genes from other moths, suggesting potential involvement in taste sensation or other physiological processes. The female-biased EoblOBPs indicated an ecological adaption related with host-seeking and oviposition behaviors. Our studies will provide the basic knowledge for further research on the molecular mechanism of gustatory perception, and enlighten a host-selection-based control strategy of insect pests.

## Supporting Information

S1 Fig (TIF)

S1 Table (DOC)

S2 Table (DOCX)

S3 Table (DOCX)

S4 Table (DOCX)
