# Supplementary figures and images for: Identification and Comparative Study of Chemosensory Genes Related to Host Selection by Legs Transcriptome Analysis in the Tea Geometrid Ectropis obliqua
Source: PLoS One. 2016 Mar 1;11(3):e0149591. doi: 10.1371/journal.pone.0149591 (PMC4773006; doi:10.1371/journal.pone.0149591)

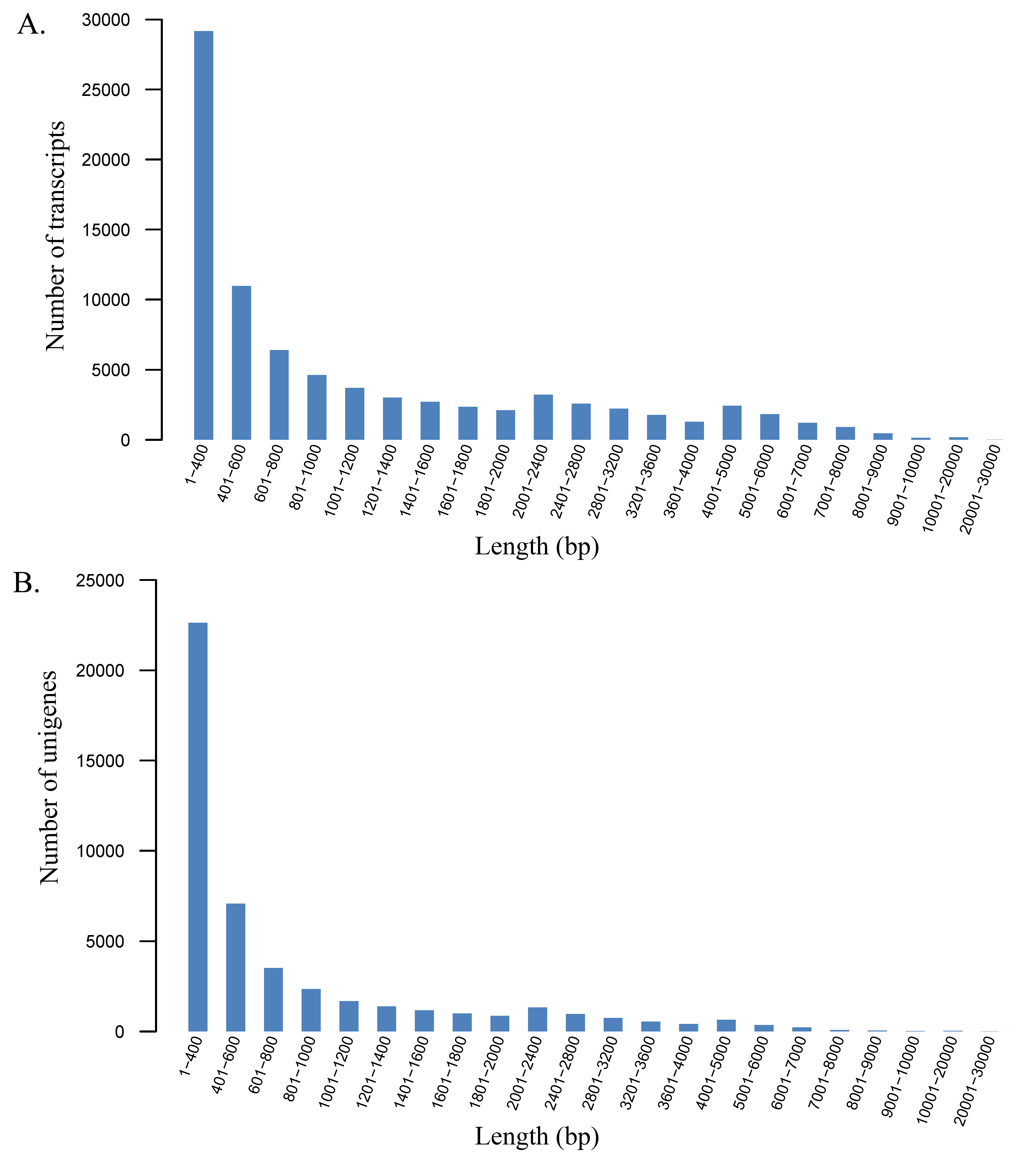

Supplement: S1 Fig — (TIF) [file pone.0149591.s001.tif]
