# Supplementary material for: Identification and Comparative Study of Chemosensory Genes Related to Host Selection by Legs Transcriptome Analysis in the Tea Geometrid Ectropis obliqua
Source: PLoS One. 2016 Mar 1;11(3):e0149591. doi: 10.1371/journal.pone.0149591 (PMC4773006; doi:10.1371/journal.pone.0149591)
Supplement: S1 Table — (DOC) [file pone.0149591.s002.doc]

| **Genes** | **5'-3'** |
| --- | --- |
| GR1 3'RACE | CCGTGCGTCGTGATCATCGTGTCTTACTC |
| GR3 5'RACE | GAGACGTTCACGCTTGGAGTAGACGGAGA |
| GR3 3'RACE | GTACCTCATCACGAGGTCTGTGGCCGTGT |
| ORco 5'RACE | GCGCTCAGTTCCATTAGCGGCTTCATGATG |
| ORco 3'RACE | GCCAATGGTACGACGGCTCTGAAGAAGCG |
| OR1 3'RACE | CGTGTGGACAGCGGTTACTATAATTCTCACAACAG |
| OR2 5'RACE | CCTCCGAAGCAGTCATTAACTTCTGGCAAAG |
| OR2 3'RACE | GACTACCAATCTTCATCAAACGTCACTCCATGA |
| OR3 5'RACE | CCAGTAGCACAATCACAAACACAACGCAGCA |
| OR3 3'RACE | GGATCCTACAATTGGCTTTGTATGGGTCTCCT |
| CSP2 5'RACE | CTAGGCATTAGCAATGGCAGCATCGAAGG |
| PBP2 5'RACE | GCTTCGGCCAAGACCTCCGCGATGAT |
| PBP4 5'RACE | CTTCTCCCACGACCATGTCCATGGACGG |
| OBP13 3'RACE | CAATCCTGGTGCTGCTTTGGCAGTATGTG |
| OBP21 5'RACE | CACCGTCAGTTACTGGCTCGTCATTCACC |
| OBP21 3'RACE | CTGACGTTAGTTTTGCTGTTAGTTGGTGCTTGC |
| OBP22 5'RACE | GTTGTCGATGAAACAACGAGCGAGCAGCT |
