# Supplementary material for: Identification and Comparative Study of Chemosensory Genes Related to Host Selection by Legs Transcriptome Analysis in the Tea Geometrid Ectropis obliqua
Source: PLoS One. 2016 Mar 1;11(3):e0149591. doi: 10.1371/journal.pone.0149591 (PMC4773006; doi:10.1371/journal.pone.0149591)
Supplement: S2 Table — (DOCX) [file pone.0149591.s003.docx]

| **Genes** | **Forward (5'-3')** | **Reverse (5'-3')** | **Forward (5'-3')** | **Reverse (5'-3')** |
| --- | --- | --- | --- | --- |
|  | **RT-PCR** | | **qRT-PCR** | |
| OBP3 | CTGGTGCTTCTCAGGAGGAA | TCGTCGTTCACAGAAGAGCA | TTGGGAATTGCTTCTGGTGC | GTCAAACGTGTCCACTGTCC |
| OBP4 | GTGCGATATCTGCAGACACC | ATGAAGTATCCCCGCTCGTT | ATGCCCACACAGAAGAAACG | ACAGCCATGAAGTATCCCCG |
| OBP5 | TTTGTGGTGGGCGTTTTAGG | GACACGGGAGCATCATTGAC | GCAGAACCCAATGACAGCTG | GACACGGGAGCATCATTGAC |
| OBP6 | GAGTACTTGCCGTCTTCAGC | CAACTTCGCCCTCTCACAAC | CGAGGTGGCTAAATCGGAAC | TCCTTGTGCTCAGTCAGACA |
| OBP7 | CTGCCGATCTAATAGGACAACC | CCTGAGGTTCTTTTCCACGC | TGCGTGTTTGAGAAGTCTGG | ACCCAAATACTGCCTGAGGT |
| OBP8 | ATGCAGGCTTCAATATTGTTTGG | ATGCAGGCTTCAATATTGTTTGG | GCAGAACCCAATGACAGCTG | GCAGAACCCAATGACAGCTG |
| OBP9 | CTACGGTGCCTGTTTTCCTG | ACATTTATCTTTGCCCGGCG | GCAGAACCCAATGACAGCTG | GCAGAACCCAATGACAGCTG |
| OBP10 | TTGTTACTTCTGGTCGGGCT | GGGACTTCGTTCTCTCCCAA | CCTAGCTTGCGTGATGAAGG | AGCCAAACTGTGACGCATTT |
| OBP11 | AGTCTATAAGGCACGGCGAA | AAGCCTTCATATCAGCCGGA | GATGCGATGACCAAAGAGCA | GATGCGATGACCAAAGAGCA |
| OBP12 | AGCCTGCTTCTGATACTTCCA | ATTGTACGCCATTTCGCAGT | TCGCTCAATGAGACCGGAAG | TATTCTCTCGTTGGTCCCCG |
| OBP13 | TCAATCCTGGTGCTGCTTTG | GAAGCCTCGCATATGTCGTC | GGCTTGCGTTTACACCATCA | GAAGCCTCGCATATGTCGTC |
| OBP14 | TAGTGCCATGACAGCGGAG | AGCCAAACTGTGACGCATTT | CCTAGCTTGCGTGATGAAGG | AGCCAAACTGTGACGCATTT |
| OBP15 | AAACAACTTAGGAGCACGGG | TGGTGGTATGGAACGATGCT | AACTGAACTACGAGGCTGCT | CGCTGCAGGGTTGTTATTGT |
| OBP16 | CTTTGATTGTCCTCGCCGTC | ACAGGCGTCGATGATCTTCT | AACCAAACCTGACGAGCAAC | ACAGGCGTCGATGATCTTCT |
| OBP17 | CGCGTCCCAAAAGTCAAAGA | ACGTCCAAATCGAGCTCCTT | ATGATGCTGCACAACTCACC | ATGATGCTGCACAACTCACC |
| OBP18 | TTATGGCTGCCGGAGTAACT | AGTACAGCTCTATCGCAGCC | AAAGGAATGTTTGACCCCGC | AAAGGAATGTTTGACCCCGC |
| OBP19 | CAGTGGCAGTATGGGAGAGA | TGTCTTCCGCCTCTGATTGT | AAAGGAATGTTTGACCCCGC | AAAGGAATGTTTGACCCCGC |
| OBP20 | CAGCCATCACTTTACCACCC | TGATACAGTATTCTTCAGGGTCGT | CAGCCATCACTTTACCACCC | CCTTTTCCCATCTGCTGTCA |
| OBP21 | GCTGTTAGTTGGTGCTTGCT | ACTGGCTCGTCATTCACCTT | TCAGACAGCCCTAATGCCAA | ACTGGCTCGTCATTCACCTT |
| OBP22 | CCTGAATGCGCTACCTGTTC | ATGAAACAACGAGCGAGCAG | CCTGAATGCGCTACCTGTTC | ATCATCCGCTCCATGTACGT |
| PBP1 | TACCCTGTCTTGGCGCATTT | AGCTGCTTCGCTAGATCGTC | ATGGCGCTGATGACGATCTA | ATAGACGGAGCCCACTTCAG |
| PBP2 | AGAGCTCGATCTTCCAGACG | AGACCTCCGCGATGATAAGG | ACAAGAGTGCAGTCGTCTCA | AGCCGGTTCTTCACTTCGTA |
| PBP3 | ATGTGGTGGAAGCTCGTGTT | AGGAGTGGATGATGCTGACC | ACTGGAAGCTGGAGTTCTCC | AGGAGTGGATGATGCTGACC |
| PBP4 | AAGGCTGAGCTAAACCTCCC | CAGCCAAAACTTCTCCCACG | ACCAAGCTCAACCTCATCGA | AACTTCTCCCACGACCATGT |
| CSP1 | CCTTCGTGGTACTGGCATCA | TCATCTGTTTGTTTTCCTTCAGG | CACTGTGCTTTCTCGGACG | GGTCTGAGCGCTTCTGTAGT |
| CSP2 | CCTCCGAGGCCTATTACACC | CAATGGCAGCATCGAAGGC | ATATCGAGGCGTTGGTGTCA | GGCTTCATAGTCAGCTGGGA |
| CSP3 | CGTTTGTTTGCTCGCCATG | CGCACAGTTTCTTCCAGTCC | TTCGGGATCCTTACCTCAGC | CGCACAGTTTCTTCCAGTCC |
| CSP4 | GGTATACCTCATTACCCTTGCG | ACGCCTAGTTCCTTCCACAA | CAAATCCTCGAGTCACAGCG | ACGCCTAGTTCCTTCCACAA |
| CSP5 | TAAGTGTGTTTTCGGTGGCG | GACTTTTCTTCGTCGGAGGC | TAAGTGTGTTTTCGGTGGCG | CGGTGCACTTTTCACAGTCA |
| CSP6 | GCGTGTTCTGGGGAGACTTA | TAGCCTTTTCCAAGTTGCCG | TGGTTGCTGACTTACCGACT | AAAGCAGCATAGTCAGCTGG |
| CSP7 | ATGTCTGTGATATCAGTTGTCAGTTTCG | TTAAGCAGACGATGTAACTTTTTCAAAC | ACCCGAGACTGCTGAAGAAA | CTTGGTGTAAGTCCGGTAACA |
| CSP8 | TGTGCATCCTTAGCTTTGCG | GCCTTAAGTTCAGCCTCATGT | CGACCTTGACGAGATCCTCA | CTGAGCCTCCGTGCATTTAG |
| CSP9 | AGTTTACACACCCTGGCTCA | TGTGCGAGTAGAGCCTCTTT | CCTCCTCATCGCTGTCCTTA | TTTAGTTCCTTCCCCTCCGG |
| CSP10 | AGTTTTCGCTTGTCTGGTCG | GTCGGGTCGTTCTTCTTCAC | AGTTTTCGCTTGTCTGGTCG | TTAAAGTCGGCTCCCTCAGG |
| CSP11 | ATGAGATCATCAATGTTGTTGTGTG | TCACAAATTTTCTGTAGCTTTTGTAGTG | GCGTTTGTTAGTGGTGACGA | GGTCCACACGCTGTTTTCAA |
| CSP12 | ATGAAGTGGACTGTGCTGGCC | TTAGAATCCGGTGACAAGAAATTTG | AACGAGTTCTGCCAGAGACA | CTCTGTTGGTTTGTTCGGGG |
| CSP13 | TTCTGTATCGTCGCTTTGGG | AGCTAAAAGACCCGGGAACA | TTCTGTATCGTCGCTTTGGG | GCGAGGGGAAAGTCAGTTTT |
| CSP14 | ATGCAGATCAAATACGCTACGATT | TTATCCAGCGTATTGGCGTACTAT | CAGAGGCAGCTTAAGTGTGC | TGTTGAGGGAAGTTGCGTTG |
| CSP15 | ATGAACGCGCTACTATTACTGGC | TTAGTCTTCAGCAAGAACAAAAGCA | GTGGTGTGCTACGACGAAAT | CTGAATCGGCGAACATTTGG |
| CSP16 | AGTCCTGGTATTCTGCGTCA | TTCTTGACGAGTTCCTGCCA | ATGACAACTTCGATGCGCAG | TTCTTGACGAGTTCCTGCCA |
| CSP17 | TCCTGGTGATTTGCTGCTTG | GTACTTCTTGCGCCATTTGC | TCCTGGTGATTTGCTGCTTG | ACTTCTCGCAACCAGTCTCA |
| CSP18 | ATGAAGGTGATCAGCTTCTACATGG | TTAGGCCTGAAGTTCCTTGAGC | CCGCGAACCAAATGACGTAA | TGCCGTCATATGGTTTTCGG |
| CSP19 | TCCCTGAAGCGCTAAGAACA | ACCACCGAACCTGTTGAGTA | TCCCTGAAGCGCTAAGAACA | CACTACGCCAGATCCAGGAA |
| CSP20 | CAGTTCTGGCCAGTGACAAA | AGCTTGCTGATATTTGCCTTCA | CTCGGCTCCTGAACTCCTAC | AGCTTGCTGATATTTGCCTTCA |
| CSP21 | CGCCACAGTATTATGCGTGT | CCGGGTCGTATTTGTTGGTG | GGCTCCTGGTACCCTACATC | CCGGGTCGTATTTGTTGGTG |
| SNMP1 | GCAGCCGATCGTAAAGGAAG | TTCTGTCCTAGCGCAGTTGA | TCAACTGCGCTAGGACAGAA | ACCCACTTCCATGACGTTCT |
| SNMP2 | TGTACTAAACCTGCACCCGT | TTCTTCTTGGAGCGAGGGAG | TGTACTAAACCTGCACCCGT | GCCTGTCACTGGGTCTATGT |
| ORco | TCCAAGGCTACCACTGAAGG | CGTCATGCCAAAGTCTTGCT | CTGAGCGCTTCGTTGGATAC | GGCCATTCGGATTATTCGGG |
| OR1 | TCAGCCACCAGTGTCTACAG | ACATGACTTGCAGCGTTGTT | AACAACGCTGCAAGTCATGT | TCGGATCGCGTCTTGGTAAT |
| OR2 | GTACCACATGCTCGCTGAAG | CTCCCATCCACAGCTGTACA | AACTTTGTCCCGAACTTGGC | TCTTGATGAAGTCCTCGGCA |
| OR3 | GTCTTTCATTCGACGCTCCC | TTGTAAAACCACACGCCCAG | CTGTGAATGTACTGGGCGTG | CACCATTATCCACACGCAGG |
| GR1 | TCAACTAGGACTGGACGAGC | AGGATGGAGGCTATGACGTG | GTAAGCTGAGCGGACAAACC | AGGATGGAGGCTATGACGTG |
| GR2 | AAGCTTGTTTGGCCTAGCAC | GAGTACCTTCCACCCAAGCT | GCTGGATTTCATGGGCAGAG | CTGTCCTCTTCGCTTGCATC |
| GR3 | AATCTGGCGCTTTGGATTCC | TAGCATGGACTAGAGCTGGC | ACGACACTGCTACACGGATA | TCCGTTGACTTGGTCCAGAA |
| β-actin | TCTTCCAGCCCTCATTCCTG | GGGCTGTGATTTCCTTCTGC | TCTTCCAGCCCTCATTCCTG | GGGCTGTGATTTCCTTCTGC |
