# Supplementary material for: Identification and Comparative Study of Chemosensory Genes Related to Host Selection by Legs Transcriptome Analysis in the Tea Geometrid Ectropis obliqua
Source: PLoS One. 2016 Mar 1;11(3):e0149591. doi: 10.1371/journal.pone.0149591 (PMC4773006; doi:10.1371/journal.pone.0149591)
Supplement: S3 Table — (DOCX) [file pone.0149591.s004.docx]

>DmelOBP28a

MQSTPIILVAIVLLGAALVRAFDEKEALAKLMESAESCMPEVGATDADLQEMVKKQPASTYAGKCLRACVMKNIGILDANGKLDTEAGHEKAKQYTGNDPAKLKIALEIGDTCAAITVPDDHCEAAEAYGTCFRGEAKKHGLL

>DmelOBP56h

MKFTLFCIALAAFLSMGQCNPDFRQIMQQCMETNQVTEADLKEFMASGMQSSAKENLKCYTKCLMEKQGHLTNGQFNAQAMLDTLKNVPQIKDKMDEISSGVNACKDIKGTNXCDTAFKVTMCLKEHKAIPGHH

>DmelOBP76a

MKHWKRRSSAVFAIVLQVLVLLLPDPAVAMTMEQFLTSLDMIRSGCAPKFKLKTEDLDRLRVGDFNFPPSQDLMCYTKCVSLMAGTVNKKGEFNAPKALAQLPHLVPPEMMEMSRKSVEACRDTHKQFKESCERVYQTAKCFSENADGQFMWP

>DsecOBP57d

MPGKMSLRFLPHLACIIFILEIQFRNSECNDPCPHNEGIDEDIAEAILSDWPANVDLTSVKRSHKCYVTCILQYYNIVSTSGEIFLDKYYDTGVIDEFAVAPKINRCRYEFRMETDYCSRIFAIFNCLRQEILAN

>DsecOBP57e

MLDRLTLYLLINFLCANVLAYTSVFNPCVSQNELSEYEANQVMEKWPDPPIDRAYKCFLTCVLLDLGLIDERGNVQIDKYMKSGVVDWQWVAIELVTCRIEFSDERDLCELSYGIFNCFKDVKLAAEKNVSISNGK

>DmelOBP49a

MLSKSQLLLLVVGFCLNAAVSADVDCSKRPSFVNPKTCCPMPDFVTAELKQKCIKFDMTPPPPPDGEASGSFESKRRHHHPHPPPCFFSCIFNETGIYQNRKLDEAKLNAYLQEVFEDSSDLQTTATQAFTTCATKVADFEANLPPRPAPSPPPGFPMCPHDAGHLMGCVFRNMMKNCPDSIRNDSQQCTDMKEFFTKCKPPRGPPPSAEDM

>EoblOBP3

MAKLFLFVVGVAVSLGIASGASQEEITAVRTAIRPFLDECGAEFGITRDQLLAAKAAGTVDTFDPCFYACFFKKIGFIDAKGLFDANVALEKNKKYFKAADDIAKIEQMGKTCSSVNDESVSDGDKACERSKLLLKCFLKEKANFTPFESS*

>EoblOBP4

MVRKFSGLLCCLCVFGISLSDSAISADTEQRCKNPPTAPQKIERVITLCQDEIKLSILREALDVIKEEHTMPTQKKRNKREVPFTHDEKRIAGCLLQCVYRKVKAVDGYGFPTLEGLVGLYSDGVNERGYFMAVLEASRECLMRSHDHFSRTVPMDNGRNCDVSFDIFECISDRIGEYCGNAGL*

>EoblOBP5

MLKLLGFFFVTWTFVVGVLGAEKLNDLKQEYDEILKECIAQNPMTAEDVESLSKDKRTYNVNCIFACALKKGGMMDDDGNLSVEGVRKSAEAYLSDDPELLKKSELFTDACKSVNDAPVSDGKKGCDRASLIFQCSVEKAPSFQLF*

>EoblOBP6

MAKLQCFVFLGVLAVFSVASAAISEEDKNAIKAEMLPVLAECGKEHGVTEKDVKEAKESNNVDAINPCFIACFMKKRKIIDDEGKYAPEVAKSEHAKYIHDAELVAKLDEISDNCASVNDQAVSDGAKGCERAKLLTACLTEHKDILTEIFKD*

>EoblOBP7

MKILFCFVLLTYTSGDLIGQPRNNKEATLKPISTCCDIPELGDPKPLAECSNPKLPGPCNDVQCVFEKSGFLIDKSTLNKETYKAHLRQWAEKHKDWSAAVERAIEDCVEKNLRQYLDIPCTAYDVFTCTSIAMLKKCPASSWKC*

>EoblOBP8

MQASILFGLVFVAAGINAGSVHLEGAQKDKAAETAMQCMKETGVKPEMVAEVKKGRLSEDEDLKKFTLCFFQKAGIISPDGKLNVDVALSKLPAGVDKTEAEKLLNDCKTKKGKTAADTAYEVFKCYQAGTKTHILL*

>EoblOBP9

MTTATVPVFLALIAVAYCGKDKPVFTDEMNEIIQTIHDACVGNTGVSEEDITNCENGIFKEDPKLKCYMFCLLEESSLADEDGVVDYDMLLSLIPEEYYDRTSKMILGCKHEDAPGKDKCQSAFDVHKCSYQKDPDLYFLF*

>EoblOBP10

MVGLGGLLLLVGLQIIASQEQGPPHGPPPQWANHKCAGPPPAIKNPQKCCEIQQMFTEEEMASCGINKFEEENRQGPPKPPDCNKQECLLKSKDCLNDDGSINHKAVAEHLNNWASEEWKPAVEAAVAVCLGENEVPGPPHICEANRLMFCIGGVIFSECPTWQDNDDCKQLKEHINECKAAKFPPPN*

>EoblOBP11

MEKSYWVVLITAIMIAGDCDAMTKEQLRKTGKMLRKQCLGKVGVEEEKISQIEKGKFIEEKDVMCYIACVYQMTQIVKNNKLSYESALKQVDLMYPADMKASVKASIENCKDVSKKYKDVCEASYWTAKCLYDDNPKDFMFA*

>EoblOBP12

MNYTLLCLSLTITSVSLLVTCSKLSTETTKVTATTESSNNMKPDDNNKELDDSKPASDTSRIGIDTKYTSNDTNALYDEVMDVLTTCNESFRIEISYLVSLNETGSFPNETDKTPKCFLRCVLQSLEVASMDDGKIDPKRAAEVFGDQRENIEETATLCAQRDEKCHCEMAYNFLKCLFSTKIENVEKSKT*

>EoblOBP13

MWSSKINPGAALAVCVFLLQMLESHAMTRQQLKNSGKLMKKTCMPKNDVTEEEVGSIEQGKFIEDRRVMCYVACVYTITQVIKNNKLSYEAVIKQVDMMFPPEMRTAVKAAAENCKEIAKKYKDDICEASYRTAKCMYEYDAENFVFP*

>EoblOBP14

MFRLVGFICVCAALTPYLASAMTAEQKQKIHEHFETIGMKCMKDHHITEADITDLRAKKVPSGPEAPCFLACVMKDIGVMDGNGLIQKETALELAKKVFEDAEELKMIEDYLHSCAHVNTEPVSDGDKGCDRAIIAMKCMIENASQFGFEL*

>EoblOBP15

MTNSTTFSKYSVMFTSFIFLTILTLSATMTMKQLRSTGKMMRKSCQPKNNVEDEKIDPIADGVFIEEQEVKCYIACIMKMANAIKNGKLNYEAAIKQADLLLPDEIKEPAKESITVCRKVSDQYKDICEASFHTTKCIYNNNPAAFYFP*

>EoblOBP16

MKTLIVLAVCFVAAQALSNEQKEKLKKHKTECLAETKPDEQLVNKLKTGDYKTENEPLKKYALCMLIKSELMTKDGKFKKDVALAKVPNAADKPAVEKIIDACLANKGNTPQQTAWNYVKCYHEKDPKHPIFL*

>EoblOBP17

MYKFGLVCFVLAASVVLKNDAAQLTASQKSKIYGSVLSAGMECMRDFPLSLDHIQAFRNKKAPNDEVAKCFTHCLYKKLGLMDDSGKISEKTAKAATKKVFKEGDEMFTKVEELISRCIHVNDAETSDGDKGCDRAKLAFECFIEHAKELDLDVDL*

>EoblOBP18

MMRYVCVIAVILAGFMAAGVTDEEKKQMHDYLVSATESCSKEFGIPSEDFEKAKRNKELQSLDPCFVACILKGNGLIDDKGMFDPAKGTSIAEKFIKSPDDIAKVKKISDICSSVNDEAVNDGDKGCDRAVLLLKCLMENKSLVV*

>EoblOBP19

MKDQISVINSTDYDYDGYGSGSMGEKFVNSMPKAADGRYYPYANGTNRTRRSEPLFSKPDNEQCLSQCVFANLQVVDSRGIPREAELWNKIQSSVTSQQSRAALKDQTSACFQELQSEAEDNGCSYSNKLERCLMLRFSDRKPSGTQTNNKQGTK*

>EoblOBP20

MFKILLFICAVSAVNCEIVRTAITLPPEIAFDIAKAIKEVCVPEDRVPDIIRMIREGETNNNTEFKKIIHCVIKEAKYMTADGKRINVEKAASIFPNKVLMFKILSQCDKNIVTNDPEEYCIKFYDCFQENTPYRLSF*

>EoblOBP21

MISQLTLVLLLVGACYGRTDLEVKGWFFSLAVICNKDYTIAPEELAMMQDHRISDSPNAKCLMACIFKKADMMDDKGNYDLEKTNKWVETEFSDSATRLESARNLFNMCKKVNDEPVTDGEKGCERAYLLSKCLVENSPKIGFATIE*

>EoblOBP22

MARFSFVAFLGVAAVINIALAITEDEKNNIRLNALPVLTSCAQELGIKMEDVVAARQAKNLDALNPCYYACFFKKINVIDNDGLFVPAVAKANHQKYVHGADDLARLSASADTCTSVNDQAVTDGANGCDRAKLLARCFIDNHGVGPFSA*

>EoblPBP1.

MARFTLSWRILALFAVFLAQIEERECSQEVMHKITKDFAYVLEDCKKQENVGDHIMQDIFNFWHEEYALVNPELGCVMLCMAGKLDLMDGDDMHHGNAHEFAKKHGADDDLAKQLVTMIHDCEKASASIADRCARALETTKCFRGKIHGLKWAPSMRVIMEEVMADMNV*

>EoblPBP2

MTKLKELLLVLVISVITRVQSSQDVMKSLTLNFGKPMEVCKKELDLPDAVTKEFLNFWREGYEVKNRLTGCAIICMSEKLELLDEGLKLHHGNAKDFAKKHGADDGMAQQLVDMIHSCMESTPPNTDPCMKTVDVAMCFKLKIHDLSWNPDPDLIIAEVLAEA*

>EoblPBP3

MWWKLVFVVVVGSAVVGTTEAADAMKLLASGFISVLEICQKELNIEDGLISDLYHYWKLEFSMMQRDTGCALICMTKKLELLTDDGKFHHGVTKEFAMKNGADDNLATEMVSIIHSCETKSEGLDDECLRALEVAKCFRVALHDLHWEPSPDVVITEVLGEM*

>EoblPBP4

MAKYHFNKSLVLNVFLTVFLYFNYGVDADSNIMKNLSLKFGEAMSICKAELNLPDSINEDFYNFWKPDYELQHRETGCMIHCLSTKLNLIDPEGKLHHGKAKEFAMSHGADEGMAQQLIDIIHNCENSTPQNEDGCLMVLAVAKCFKVEIHKLNWTPSMDMVVGEVLAES*

>BmorGOBP1

MWKLVVVLTVNLLQGALTDVYVMKDVTLGFGQALEQCREESQLTEEKMEEFFHFWNDDFKFEHRELGCAIQCMSRHFNLLTDSSRMHHENTDKFIKSFPNGEILSQKMIDMIHTCEKKFDSEPDHCWRILRVAECFKDACNKSGLAPSMELILAEFIMESEADK

>BmorGOBP2

MFSFLILVFVASVADSVIGTAEVMSHVTAHFGKTLEECREESGLSVDILDEFKHFWSDDFDVVHRELGCAIICMSNKFSLMDDDVRMHHVNMDEYIKGFPNGQVLAEKMVKLIHNCEKQFDTETDDCTRVVKVAACFKKDSRKEGIAPEVAMIEAVIEKY

>BmorPBP1

MSIQGQIALALMVYMAVGSVDASQEVMKNLSLNFGKALDECKKEMTLTDAINEDFYNFWKEGYEIKNRETGCAIMCLSTKLNMLDPEGNLHHGNAMEFAKKHGADETMAQQLIDIVHGCEKSTPANDDKCIWTLGVATCFKAEIHKLNWAPSMDVAVGEILAEV

>BmorPBP2

MKLQVVLVVLTVEMVCGSRDVMTNLSIQFAKPLEACKKEMGLTETVLKDFYNFWIEDYEFTDRNTGCAILCMSKKLELMDGDYNLHHGKAHEFARKHGADETMAKQLVDLIHGCSQSVATMPDECERTLKVAKCFIAEIHKLKWAPDVELLMAEVLNEVSWKS

>BmorPBP3

MARYNIVVAVLVLGVVGARGSSEAMRHIATGFIRVLDECKQELGLTDHILTDMYHFWKLDYSMMTRETGCAIICMSKKLDLIDGDGKLHHGNAQAYALKHGAATEVAAKLVEVIHGCEKLHESIDDQCSRVLEVAKCFRTGVHELHWAPKLDVIVGEVMTEI

>BmorOBP5

MKQRLRVLLLRFCILQTVLSESGVDVVKNLSLSFARFFLECDEERHFQPEVRLKVMTFWYSESSTWDRDVGCAFLCIFKKMEIDNPQDPSYRTHLELLSFANSEDNKIANQMVEIFYACGENTETDPCLWALEQVKCYKNRINQLGLTPTF

>BmorOBP7

AVTEEELKIEFTKLVMKCTKDHPVDMSELMQLQQLIAPKKTESKCLLACAYKLNGVMTSQGLYNLEHAYKIAEMSKNGDEKRLENGKKVADICVKVNDVEVSDGE KGCERAALIFKCTLENAPKVFKFGSSEYNCQ

>BmorOBP8

MLRVVVICVCFLVIAPYGINASSLDDLKMVYKNVIKECVGDYPITAADLKLIKARQIPNDDIKCVFACAYKKTGMMTEEGMLSVEGIKDMSQKYLSDNPEQLRKSKEFAEACSSVNDQQVSDGTKGCERAALIFKCSTEKITNFGFEL

>BmorOBP9

MLRVVVICVCFLVVAPYGINAVSYEQKIKIRDQLDRAGFECFKDHKITEDDIKNLRANKPATGENVPCFIACVMKKTGVMNDQGVIRKGPVLELAKKVLADDKDIKKLQDYIHSCSHVNSETVHDKGKGCEFAMQAYTCMSANASKFGFNI

>BmorOBP10

MLRVVVICVCFLVIAPYGINAVSDEQKIKIREQIDKSGFECFKDHKITEDDIKNLRARKPATGENVPCFIACVMKKTGVMNDQGVIHTEPVLQLAKKVLTDDKDIKKLQDYIHSCS HVNSKTVHDKGQGCEFAIQTYTCMSANASKFGFDV

>BmorOBP11

MSANSFVVLAFCALAVGVNALTEEQKAEITKSSLPLIAECSKEFSVNQGDIDAAKKLGDPSGLNSCFVGCFMKKAGIINASGLFDVAATIEKSKKYLTSEEDLKAFEKLTETCAPENDKPVSDSDKGCERAKLLLDCFVANKGSFSVFSL

>BmorOBP12

MTSFMVFFVLSVLTLKYSDALTDEQKNKIQSKFIEIGAECIVEHPISIDDINSFKNKKFPSGVNAGCFVACIFNKIGLFDDKGNLSHNSALEKAKGIFNADEEVKNLEEFLNRCAKVNGEAVGDGVKGCERAKLAYNCLIENSLEFGFNIDF

>BmorOBP13

MLKIHVLLCFGMAILYFGSAKAVTPEESKAFEAFAKPVIEQCQKDFGMDKESFAQKNLDEIDECLIACVVEKFGITNDEKIDGDALKALVTKFVGNEEERNKINKIVEECTEDANKSGDGTCNTSTILFLCLLKNGKDLWGF

>BmorOBP14

MSRQQLKNSGKMLKKQCMGKNDVTEEEIGDIEKGKFIEQKNVMCYIACIYQMTQIIKNNKISYEASIKQIDLMYPPELKESAKASAGRCKDVSKKYKDICEASYWTAKCMYEDNPKDFIFA

>BmorOBP15

MFLKNIFIECVLLYFVMLNTSFVNTMTKQQIKNSGKILKKACISKNDVTEDQISDIDKGKFIEDKNVMCYIACVYSMSQVVKNNKFVHDAMVKQVDMMFPTEMRDAVKASIANCRGVAKNYKDICEASFWTAKCMYEFDPANFVFA

>BmorOBP16

MRISFLFLISVTIITFDSVFAMTRAQVKKTMTIMKNQCMPKNGVTEDQVGKIEEGIFLENHNVMCYIACVYKTIQVVKNDRLDKDLISKQIDVLYPQEIRESTKKAVGDCINLQEKYDDWCEGIFRSTKCLYEKDPANFIFP

>BmorOBP17

MTRQQLKNSGKIMKKTCMPKNDVTEEEIGQIEQGKFLEQRNVMCYIACIYTVTQVVKNNKLSYDAVIKQVDVMFPAEMRPAVKAAAENCKDISKTFKDICEASYWTAKCMYDFDPKNFVFP

>BmorOBP18

MILIVIAKFLILISLCETMTMKQIKNTGKMMRKSCQPKNNVDDEKINPINDGVFIEENEVKCYIACIMKMANTMKNGKLNFEAAMKQADLLLPDEMKEPTKEAIVACRKVADSYKDVCDASFHVTKCIYNHNPSVFFFP

>BmorOBP19

MTSAKTDVEIKAWFLGQAVECSKDHPVTTEELRMHKHELPDSKNAKCLMKCVFRKCNWLDSKGMYDINAAYASSTKDFSDDKTKQENANKLFDTCKSVNEENVGDGEEGCDRSLLLAKCLTKAAPQVSIYYS

>BmorOBP20

MAVHIFLILASYMALAAHGQLDDEIAELAAMVRENCADESSVDLNLVEKVNAGTDLATITDGKLKCYIKCTMETAGMMSDGVVDVEAVLSLLPDSLKTKNEASLKKCDTQKGSDDCDTAYLTQICWQAANKADYFLI

>BmorOBP21

MITASLHVIFALLAFVYGGKDKPVLSEEIKEIIQTVHDECVGKTGVSEEDITNCESGIFKEDVKLKCYMFCLLEEAGLVNDDGTVDYEMFTSLIPEEYFDRATKMIFSCKELDTPDKDKCERAFEVHKCSYEKDPDFYFLF

>BmorOBP22

MLKVFVVVVCTLGASQLCAALYTQKVAVSFPKDKTTIVVEAMKSCIAKTGANPNVIEVISSGKVSEDEKFKEFFYCACNDIGVVNPDGHIKVKECIELFPKETQPLVEPVI KNCDKEGVNKYDTLFKYLKCFQETSPVRVTLA

>BmorOBP23

MTSKVLLSCVVLAVLATTVLAEDSRKLVSFAPEVAKKLKVLIQECLNENGLGEDAIEVIRAGEYREDEPFQNLVYCAYKKFGALDENNRIISQVAAASFPKDIDVVTVIESCGKEDGNTPVEQVFKYFKCFQKNSPVRMQLY

>BmorOBP25

MKSVVLICLAFAVFNCGADNVHLNEDEREKANWYTAECGVETGVSTEVINAAKIGKYSKDKAFKKFVLCFFKKSAILNSDGTLNMVVALAKLPSGVNKSEAQSVLEQCKNKTGQDAADKAFAILQCFHKGTKTHILF

>BmorOBP26

MKSVVLICLAFAVFNCGADNVHLAETQKEKAKQYTSECVRESGVSTEAINAAKIGKYSKDKAFKNFVLCFFNKSAIFNSDGTLNMDVALAKLPPGVNKSEAQSVLKQCKNKTGQGAADKAFEIFRCYYKGTKTHILF

>BmorOBP27

MKSVVLICLAFAVFNCGADNVHLTETQKEKAKQYTSECVKESGVSTEVINAAKTGQYSEDKAFKKFVLCFFNKSAILNSDGTLNMDVALAKLPPGVNKSEAQSVLEQCKDKTGQDAADKAFEIFQCYYKGTKTHILF

>BmorOBP28

MLKVFIVTFFAFQLSAIARLQANGCVAVPFPKDKTIIIVEAMKSCIAKTGANPNFIDVIRSGKVSEDEKFKEFYYCTCNDTGFVNPDGHIKVKECIELFPKETQPLVEPVIKNCDKEEGVNKYDTLFKFLKCFQETSPVRVALA

>BmorOBP29

MTGPAAAAVLLALLAAAGQATTGCKNCVILGKEERAMFRSHSDACLAQSRVEPRLLESMMNGELIDDAALRKHVYCVLLSCKMIGKDGKLLKAAILGKLAARPAGRDVTKVLEACAEQPGASPEDVAWNIFRCGYNRKAVLFDYMPAGGASSGNTENHP

>BmorOBP30

MRSFVILLNYGLLCCGQFMAEDYYYDIVTRDPDDLMREKENEVRALRAFQADCAEDVQVKPDLVVNLKSGDWQTEDVSLKKWALCVLMKLGLMTAQGVFKMNEAMSKIPDMNDKIIAEKLIDDCLSLQATTPHDAAWNYIKCHHQKDPEGNFSSLNIF

>BmorOBP31

MKTFIVFVVCVVLAQALTDEQKENLKKHRADCLSETKADEQLVNKLKTGDFKTENEPLKKYALCMLIKSQLMTKDGKFKKDVALAKVPNAEDKLKVEKLIDACLANKGNSPHQTAWNYVKCYHEKDPKHALFL

>BmorOBP32

MYSHKYLNDFTNIPEILIILLSSVALMSYGYNTKLFSHSLGSEPSLSILYARDKKSDKVTNECLMEMYPKNLYKYPLRIDRNDIPCIIHCVLKKFGIISNDGFINIKNYYRRVQAIHRYDPRILISDVGETCAQNINGMNLDHDVCKKAKVFNDCTQLYAISYREPEDW

>BmorOBP33

MYAHDKLSDMIADQCLNEMYPRSKRLEIEESDEPCIIFCVLKKFGIMSPTGVINLEAYRKRVQLPEQLAQRNSINDFGSACLESAEATQHKQDVCKKAKVFNECTHLYKILLK

>BmorOBP34

MEKMILLNVFAVVLPCVLASRTRGSSGTLVDFTDPKVQGHLDALVRMAQSCVIKVRATPKDVRAYFTNSSPVSRSGQCFATCMLEQSDIINHGKVNRDLLVHLAGLVNGKNSRVVRKLNSVSRLCLDSISGMTDRCQLASTYNDCLNENMIEFAFPLDIAEEAVRKMPFHLIQPK

>BmorOBP35

GMSTHVLDFKRNMTECLKEVQNNDKRPIKRLSPKQESPIHGECLIACVLKKNGVIQNGKVNKDNLMALVSKFHAKETKLMKKLEKNLDRCINISVKNHDECSLASQLNDCTNDIMASSKQKILFNY

>BmorOBP36

MAVSEISRILTFLTIVSFIYIVYSFKPLTKDEHIERYNKMNEDIEPFRKNLTECARQVKASMADVEKFLKRIPQSNMEGKCFVACILKRNSLIKNNKLSQENLLEVNRAVYGDDSEVMSRLKTAILECSKIVEDIFEICEYASVFNDCMHMKMEHILDKITMERRMEALGQMSSNPDEWSEEEDEMLKLVKDEL

>BmorOBP37

MFYPFRFTLLFYGLFVIYLVRAEPEKENHFTLALKKTLFSTARSCMSHVNANETDLEYLRKDPPFPDKAACIIKCLLEKIGVVKNNKYSKMGFLTAVSPLVFTNKKKLDHYKSVSENCEKEINHDQTTECELGNEVVSCIFKYAPELHFKT

>BmorOBP38

MANLVLLLTFVLMTLSMARLKSTEAPKSKTALFNDQDNMGYEELDMEEIMSACNESFRIEYAYLESLNDSGSFPDETDKTPKCYIRCVLEKTEILSENGVLNPATAALVFAGERNGKPMSDLEEMAVACADRHEKCKCEKAYNFVKCLMYMEIDKYEKKN

>BmorOBP39

MVRKISALLCCFCVLGISMCDSAISTDNEQRCKNPPTAPQKIERVITLCQDEIKLSILREALDVIKEEHTMPAERKRNKREVPFTHDEKRIAGCLLQCVYRKVKAVDGFGFPTLEGLVGLYSDGVNERGYFMAVLEASRECLMKNHDKFSRTTPMDNGRNCDVSFDIFECISDRIGEYCGTSGL

>BmorOBP40

MSEFIQPSWRTQCNFRLNWDNRNRLSIDISHGAATTQTPVPTTKPKALRDFMVVPQSCDKTTCVFKKLNIVSDKGVVDVKSFIKLLDKFTNSYPVWNSAKARVITTCLRKSLIAYDGGCELNNILACTFDVLSENCPLNGNNQTC

>BmorOBP41

MLTILFLLPIVVGVLSGNIPEQPRVYCGELPNTIYSCLGNPKIIQPEVSEKCNKPISECDKTRCIFKESGWAKNNVIDKKKVSDYFEQFAKDNPDWSAAVQNFKTTCLSDSLKPQGVDTNCPAYDIIHCALISFIKFASPSQWSTSEQCVYPRQYAGACPVCPERCFAPSVPNGSCNACLALLRTP

>BmorOBP42

MMGYACVFVILAVLQAISAEDPPGLPPFLKDAPEKCKSPPRVKNPNECCISEPFFKEADFIECGIEKPGSERGPPDCSKQNCLLKKYNLLKNDETPDIEAIKSLLDKYIEKNPSFKSSVEKAKECLREDLPGPPQICLANRMTLCIGTVLLMECPDEKWNTTDDCKAFKDHMTECQKYFPK

>BmorOBP43

MKVCVLFAIFTVAQAAKATLKPISACCNIPELGNPEPLAECSNPKLPGPCKDIQCVFEKSGFLTENKTLIKEAYKTHLRQWAKEHEGWSVAVEKAISDCVDKDLRQYLEFPCSAYDVFTCTGIAMLKKCPNEHWTC

>BmorOBP44

MSRLVLFFTILVVLQEFIINLYFNFITEIDSCCVKKYPKLFDSEFITECYNTQRKANDKCERDMCVARKLNLLTEEDSINKDALLRFVEEGFKTEIDLVNAIKKKCFEEDISNIGKPEMCEVAKYKICITSRMAEDCPKWDSKGICSSAQQKVENFMKMLS

>SlitPBP1

MANARWRFVFVVYALYLTSAVLGSQDLMVKMTKGFTRVVDDCKTELNVGDHIMQDMYNYWREDYQLINRDMGCMLLCMAKKLDLMDDQTMHHGKTEDFAKSHGADDDVAKKLVSVIHECEQQHAGIADDCMRVLEVAKCFRTKIHELKWAPSIEVIMEEVMTAV

>SlitPBP2

MAFCPSVTMSLRVALVVAASLLVVVQASQDVMKNLAVNFAKPLDDCKKEMDLPDSVTTDFYNFWKEGYELTNRQTGCAILCLSSKLEILDQELNLHHGRAQEFAMKHGADEAMAKQIVDMIHTCAQSTPDEAADPCMKALNVAKCFKLKVHELNWAPSVELIVGEVLAEV

>SlitPBP3

MGSRNVFVALVVLTVGMREIEPSKDPMKYIASGFVKVLEECKHELNMNDHLIADLFHYWKLEYTLLNRDTGCAIICMGKKLDLLDASGRMHHGNAQEFAKKHGAGDEVASQIVQIIHDCEKKHERDDDECLRVLEVAKCFRTGIHELNWQPNVEVIVSEVLTEI

>SlitGOBP1

MLLLLRALPLLAAVLPLRADVNVMKDVTLGFGQALDKCRQESQLTEEKMEEFFHFWREDFKFEHRELGCAIQCMSRHFNLLTDTSRMHHENTEQFIQSFPNGEVLARQMVELIHACEKQHDHEEDHCWRILHVAECFKQACVQRGIAPSMEIMITEFIMEAEAR

>SlitGOBP2

MTSKCCLLLVLMAAATSSVMGTAEVMSHVTAHFGKALEECREESGLSAEVLEEFQHFWREDFEVVHRELGCAIICMSNKFSLLQDDSRMHHVNMHDYVKSFPNGHVLSEKLVGLIHNCEKQFDSMTDDCERVVKVAACFKVDAKAAGIAPEVAMIEAVMEKY

>SlitOBP3

MWMQALVLTLATLATLAAAAVEMDEDMAELARMVRDNCAGETGVDVALVEKVNAGAELMPDDKLKCYIKCTMETAGMMADGEVDIEAVLALLPPSLAEHNAPAL RACGTQRGADHCDTAFRTQQCWQNANKADYFLI

>SlitOBP4

MTKVLFAIVLTMITFAVVLSASTKEAMTTTMSDQVNSIDVDVLAVMDMCNDSYRIDPTYLQALNESGSFIDETDKTPKCFIRCVFENVGIVSEDGKQFNPARAAVIFAGERNGKPMEDIADMTALCATDRQETCPCDRSYKFLRCLMSMEIERYEKS

>SlitOBP5

MSVVRCSSLLVAIFCFVSVNAISGDEEAGIKDALRPFVQECADEFGITEEQFEEAKKKASAADIDPCFMSCFLKKAEFFDSQGKFDVDSTMAFAKEHLTSEPAMKFVEAVGD ECVKINDEDVSDGDKGCDRAKLLFECIAETKKKME

>SlitOBP6

MSKFTCLVLCVVAVSLSGVHATAEEKAAFIEAVKPYVQECSKEHGVTPEDIKSAKAAGNADGINSCFLSCVYKKAEVITEKGEYDADKALEKLKKFVSNEDDYAKFANIGKKCASVNEKSVSDGEAGCERAALLTSCFLEHKSEISA

>SlitOBP7

MDQKRICLFVIAMFLASGSDAMSRQQLKNSGKMLKKNCMNKIGVTEDQIGSIDKGKFIEDRKVMCYIACIYELTNVIKNNKLNYEASIKQIDLMYPPDVKESAKAAVEKCKDVQKKYKDICEASFYAAKCMYEFKPEDFIFA

>SlitOBP8

MLLTKIVKFFILVATCEAMTMKQIKNTGKMMRKTCQP KNNAEDEKIDPISDGVFIDEKEVKCYMACIMKMANTIKNGKLNYDAAMKQADLLFPDDIKEPAKEAITACRKVADAHKDICDASFHVTKCIYNHNPGIFYFP

>HarmPBP1

MEFHRSTMMSVRLALVVAVCLFIRVDASQDVIKNLSMNFAKPLEDCKKEMDLPDSVTTDFYNFWKEGYEFTNRQTGCAILCLSSKLELLDQELKLHHGKAQEFAKKHGADDAMAKQLVDLIHGCAQSTPDVADDPCMKTLNVAKCFKAKIHELNWAPSMELVVGEVLAEV*

>HarmPBP2

MAASRWLFARAFCLVLMMGSAMSSKELLTKMTGGFTKVVDACKTELSVGDHIMQDMYNFWREEYQLVNRDLGCMIMCMTAKLDLIGDDQKMHHGKAEEFAKSHGADDALAKQLVGLIHGCETQHQAIEDHCSRALEIAKCFRTKIHELKWAPSMEVIMEEIMTAA*

>HarmPBP3

MGSRHVFFALVVLAVSVRKAEPSKDAMQYITSGFVKVLEECKHELNLNEQILADLFHFWKLEYSLLGRDTGCAIICMSKKLDLLDANGRMHHGNAAEFAKKHGAGDEVASKIVTIIHECEKKHEQDGDECLRVLEVAKCFRTGIHELNWQPKVEVIVSEVLTEI*

>HarmGOBP1

MPGVLRALLVLAAAAPLLADINVMKDVTLGFGQALDKCREESQLTEEKMEEFFHFWRDDFKFEHRELGCAIQCMSRHFNLLTDSSRMHHDNTEKFIQSFPNGEVLARQMVELIHSCEKQFDHEDDHCWRILHVAECFKGSCVQRGIAPSMELMMTEFIMEAEAR*

>HarmGOBP2

MTSKSCLLLVAMATLTASVMGTAEVMSHVTAHFGKALEE CREESGLSAEVLEEFQHFWREDFEVVHRELGCAIICMSNKFSLLQDDSRMHHVNMHDYVKSFPNGHVLSEKLVELIHNCEKKYDTMTDDCDRVVKVAACFKVDAKAAGIAPEVAMIEAVMEKY*

>HarmOBP1

MSKFTFFVLCVVAVSLSKVYASDEDKAKLHEALKPLVEECMKDHEVSLDDLKAAKEAKSADGVKPCFLACVYKKAEVLNDKGEFDADHALEKLKEFVSDEDVLAKVAEVGNTCKAVNDKAVSDGDAGCERAALLTACFLEHKAEILV*

>HarmOBP2

MMDRKRLCLLIIAMFLAQGSDAMSRQQLKNSGKMLKKNCMNKNQVTEDQIGSIDKGKFVEDKKVMCYIACIFEMTNVVKNNKLNYDASIKQIDLMYPPDLKESAKAAVEKCKDVQKKYKDICEASYWTAKCMYDFKPEDFIFA*

>HarmOBP3

MSKFTCFVLCVLAVSLGEVRSNALEKAAIRAAVYPLIVDCAKEHAVTLEQLKAAKASHSAEGINPCFQSCVYKKTGIFNDNGEYDVANAKTKLQKFVTDEDEYARIAEVGKTCASVNDKSVSDGAAGCERAALLTACFLEHRAQIII*

>HarmOBP4

MSKLTCVVFAAVAVVFSNVNADDETRASFRQVLGPLVMECRNEFGITEDDLKKAQQERSPDALKPCFIACVFKKFGIITSAGKYDSDASISRIKDVVKNDDLLAKLKSVGEKCNSVNDASVSDGDAGCERAALLAKCFIENKSELSI*

>HarmOBP5

MSKFTCLVLCVVAASLSQAYASEEEKAAFREAIKPIVEECSKEHGVSHDELKSAKDNQNADNIKPCFLGCVYKKAEVFNSKGEYDVDKALEKLKKFVSNDEAYAKFAEVGKKCASVNDKAVSDGDAGCERGALLTACFLEHKAEVPL*

>HarmOBP6

MSKFTCLLLCVVAVSLSKVHATEEEKEAIRAAVRPIMQECGKEHGVTLDDLKAAKAAHSADGIKPCFQSCVYKKAGIFNDNGEYDIANAKTKLQKFVTNDEEYARIAEVGKMCASVNDKPVTDGAAGCDRAALLTACFLEHRAQIII*

>HarmOBP7

MFRFGVLSFVVLLFCMESSYALSSEEELSIKEALHPFVVECAEEYGMTEEMFEEAKKKGSAEDIDPCFMSCFLKKTGFFDDSGKFDAEKSISFAKEHITSESAIKFLEAGAGECVKINDEDVSDGENGCDRAKLLFDCLTELKKKMSE*

>HarmOBP7.2

MSRFGVLSFVVLVFCMENIYALSSEEELSIKEALHPFVVECAEEYGMTEEMFEEAKKKGSAEDIDPCFMSCFLKKTGFFDDAGKFDAEKSISFAKEHITSETAIKFLEAGAGECVKINDEDVSDGDKGCDRAKLLFDCLTDLKKKMSE*

>HarmOBP8

MLLIEIVKFLTLVAMCEAMTMKQIRNTGKMMRKSCQPKNNVADEQIDPIAEGVFNEDKEVKCYMACIMKMANTIKNGKLNYEAAIKQADLLLPDDIKEPAKEAITACRKVADAYKDICDASFHITKCIYTQNPGIFYFP*

>HarmOBP9

MCKFSVLFLYSAVMAVNIWSASCISEEDKAAIITAIAPLAQNCGSECGLDNDDFEKYKEDGSDMDPCFKACLMTQMGVLDKEGKYDGKGLHKAMEEADYPGDKDDAQKFLDELDRCFDAKGDNSGSDEEAKMKRADVLFRCMQDMKEK*

>HarmOBP9.2

MCKCSVVFLYLAVMAINIWRASCLSEEDKAAIITAIAPLAQNCGSECGLDNDDFEKYKEDGSDMDPCFKACLMTQMGVLDKEGKYDGKGLHKAMEEADYPGDKDDAQKFLDELDRCFDAKGDNSGSDEEAKMKRADVLFQCMQDMKEN*

>HarmOBP13

MFTGTLPLVVFLATFAYGGKEKPVFSDEIKEIIQTVHDECVAKTGVAEEDITNCENGIFKEDPKLKCYMFCLMEEASLVDDDDAVDYDMLVSLIPEEYVDRTTKMIFSCKHLDTPDKDKCQRAFEVHKCSYEKDPDLYFLF*

>HarmOBP18

MKSFVVFCVLVAGAFAANVSLPPKQNEKANQIATECMKESGLKPEVLAEAKKGHISDDEHLKKFTFCFFKKAGIVSEDGKLNTEVALAKLPPGVDKAEAEKLLETCKGKTGKDVTDTVFEIFKCYHHGTKTHILLGF*

>HarmOBP15

MGSRHVFFALVVLAVSVKKEKPSKHPMPYITSRFVKVLEECQHELKLNEHILEHLFHFWKLEYSLLGKDPGCAIICMSTKLDLLDLYGRMHRGNAAEFAKKHAAGDEVPSKIVTIIHFCQKKHEQDGDECLQVLEVATCCRTGLHDLNWQHQVEVIVPDVLTEI*

>HarmOBP16

MFKLCVVLAFIVATCHGGTLERTSSTCGQIPRELTACLDLQPAVSPEIQEKCRRANECERLTCVFREYNLLDGAEVNKERTAAFLDNFVKQYPSWEVAIDVAKTSCLRSSGLKPQGVFLDCPAYDIIQCVFANLVKNALPSQWSSMSQCNHAREFAAACPICPDACFAPLVPIGTCNACSAARRSS*

>HarmOBP17

MRAWSVTLVALLGALGAARAVAMDEDMAELARMVRENCAAETGADVALVERVNAGADLMPDDKLKCYIKCTMETAGMMADGEVDIEAVLALLPPELAEHNAPSLRACGTVRGADHCDTAFRTQQCWQNANKADYFLI*

>HarmOBP18a

MTRQQLKNSGKLMKKSCMPKNDVTEEEVGDIEKGKFIESRNVMCYVACIYTMTQVVKNNKLSYEAVIKQVDMMFPAEMRDAVKAAATSCKDITKKSKDLCESAYWTAKCMYDYDAENFVFP*

>HarmOBP19

ARTEHEIKEWLFREGVACNKDFPITPDEMMMLKDNKLP DSTNAKCLIACIFKKTGMIDSKGMFDPDKSIAMTEKDFADNPEKLATSKKLMEACRGVNEQAVADGEKG

>HarmOBP20

KVFYLLTVLSACYGAVDITKYFKTCNRNAIDVNDCMADAVQKGIAVMINGIDELGIPPIDPYLQKDFRLEYKNNQIAAKLNMKNIQVEGLRAAKVHDARLRADDDKFHLEVDLTSPKVTVHAEYHGEGKFNSLRILAFGEVNTTMTDLVYTWKLDGVPEKNGTETYIRIKEFYMRPDVGSIVTNFKNDNPESRELTDLGTRFANENWRTLYREFLPYAQANWNKIGTKVANKLFLKVPYDQLFPTSS*

>HarmOBP21

FQMSRAQVKKTMSLVKNQCMPKNSVTEDQVGKIEEGVFLEDRNVMCYVACIYKNLQVVKNDKLDMSLITKQIDALYPPELKEPVKKAVSLCIHSQDNYNDLCEKVFHASKCLYEKDPASFIFP*

>HarmOBP22

MTREQIKNSGKLIKKTCMAKNDLSEDQVKDVDKGKFIEEKPFMCYIACVYKMGQTIKGNTVNHDMMIKQVEMMFPNEMKAPMKAAIEHCRPVVKKYKDVCEVSYWTAKCIYEFDPPNFMFP*

>SexiPBP1

MAGAKWQFVCVVFALYLTSAALGSQELMMKMTKGFTKVVDDCKAELNAGEHIMQDMYNYWREDYQLINRDLGCMILCMAKKLDLMEDQKMHHGKTEEFAKSHGADDEVAKKLVSIIHECEQQHAGIADDCMRVLEISKCFRTKIHELKWAPNMEVIMEEVMTAV*

>SexiPBP2

MAFCRSATMSVRVALVVAASMLVVVQASQDVMKNLAINFAKPLDDCKKEMDLPDSVTTDFYNFWKEGYELTNRQTGCAILCLSSKLEILDQELNLHHGRAQEFAMKHGADETMAKQIVDMIHTCAQSTPDVAADPCMKTLNVAKCFKLKIHELNWAPSMELIVGEVLAEV*

>SexiPBP3

MGSHNVFVALVLLAVGMRVAEPSKDAMKYITSGFVKVLEECKQELNMNDHIIADLFHFWKLEYALLSRDTGCVIICMSKKLDLLDANGRMHHGNAQEFAKRHGAGDDVASKIVQIIHDCEKKHERDDDECLRVLEVAKCFRTGIHDLDWQPKVEVIVSEVLTEI*

>SexiGOBP1

MLFLLRALPLLAAVLPLRADVNVMKDVTLGFGQALDKCRQESQLTEEKMEEFFHFWRDDFKFEHRELGCAIQCMSRHYNQLTDSSRMHHDNTEQFIKSFPNGEVLARQMVELIHSCEKQYDHEDDHCWRILHVADCFKQGCVQRGIAPSMEMMMTEFIMEAEAR*

>SexiGOBP2

MTAEVMSHVTAHFGKALEECREESGLSAEVLEEFQHFWREDFEVVHRELGCAIICMSNKFSLLQDDTRMHHVNMHDYVKGFPNGHVLSEKLVELIHNCEKRFDSMTDDCERVVKVAACFKVDAKAAGIAPEVAMIEAVMEKY*

>SexiOBP1

MSKFTCLVLCVVAGCLSGVHATAEEKAALIEAVKPYIQECSKEHGVTPEDIKSAKEAGNADGINACFLRCVYNKAGVINDKGEYDADKALEKLKKFVSNEDDYAKFAEIGKKCASVTETSVSDGEAGCERAALLTSCFLEHKSEVHA*

>SexiOBP2

MKSFVVFCIVLVVGVCANEKGNKLDRPFASECIKETGVKNELLEEAKKGIISEDPAFKAFTYCFFKKIGIVGEDGLLNRDVAIAKLPSGVDKSEAEKLLDSCKSKTGKDAVDTVFEIFKCYQQGTKSHIMFAS*

>SexiOBP3

MVKLTCVVFCAVAMALSVFVAGEDANSVFQGAIKPLIAECAKEYKLSDEELLKNRGLAGLSNLPPCFIGCVLKKFDIINDKGLYDAEAGIAKIEKLLPNNEFLDKISGVLKSCESANEKSVGDGDAGCERAVLVATCYLEHKTAVIA*

>SexiOBP4

MWNFLVVFLAICSCVYGLTEEELKMEFTKLIMKCNKDGKVDMTELVQLQNYVVPTKQTTKCVLACAYKAAEVMNAKGEYDIDHAYKVAEMMKNGDEKRLVNAKKMADLCVKVNEQSVSDGEKGCDRAAMIFKCTVENAPKFGFKL*

>SexiOBP5

MTMKQIRNTGKMMRKTCQPKNNVEDEKIDPIAEGVFIDEKEVKCYMACIMKMANTIKNGKLNYDAAIKQADLLLPDDIKEPAKEAITACKKVADAHKDICDASFHITKCIYNHNPGIFYSP*

>SexiOBP6

MLGSLLFVFAFSVFSLGAEALLIDDLKQKYADSILQCSQQYPLDRADAELLQNKVMPDKESTKCLFACVYKVTGVMSDQGELSVEGVNALSQKYLADDPEKLKKSEEFTEACRTVNDAPVSDGARGCDRAALIFKCTIEKSPDFSFV*

>SexiOBP7

MTKVLFAIVLMMITFAVTLSASTKEAMTTTMTDQVNSIEVDVLAVMDMCNDSYRIDPTYLQALNESGSFIDETDKTPKCFIRCVFENVGIVSEDGMQLNPARAAVIFAGERNGKPMEDIADMTALCATDRQETCPCDRSYKFLRCLMSMEIERYEKS*

>SexiABP1

MSVVRYSSFVVALFCLVSVNAMSGDEEAGVRDALRPYVQECADEYGITEEQFEEAKKKASADDIDPCFMSCFLKKAEFFDAQGKFDVDSTMAFAKEHLSSEPAMKFVEAVGDECVKINDEDVSDGDKGCDRAKLLFDCIAETKKKMD*

>SexiOBP10

MDRKRICLFVIAMFLASGSDAMSRQQLKNSGKMLKKNCM NKIGVTEDQVGSIDKGKFIEDRKVMCYIACIYELTNVIKNNKLNYEASIKQIDLMYPPDIKESAKAAVEKCKDVQKKYKDICEVSFYAAKCMYEFKPEDFIFA*

>SexiOBP8

MARRQQGAMFTETLPLFVILVAVTHGGKDKPVFSDEIKEIIQTVHDECVAKTGVAEEDITNCENGIFKEDAKLKCYMFCLLEEASLVDDDDTVDYDMLVSLIPDEYYERTTKMIFACKHLDTPDKDRCQRAFEVHKCSYEKDPDLYFLF*

>SexiOBP9

MKTLFVFAACILLAQALTDEQKEKLKKHRTECLSETKVDEQLVNKLKGGDYKTESEPLKKYALCMMMKSELMTKEGKFKKDVALAKVPNPADKPTVEKLIDACLANKGNTPHQTAWNYVKCYHEKDPKHAIFL*

>SexiOBP11

MKEGNRYSHERRITNDSGDQLMVINATDDDYSGYGSGNMGEKLLTSVPRPASSSNNINKNNTRRTRRNEPFLNRPDSDQCLSQCVFANLQVVDSRGIPREAELWNKVQTSVTSQQSRSALHDQIRACFQELQSEAEDNGCSYFNKLERCLMLRFSDRKVDGKGNPKKSSTEQT*

>AipsPBP1

MAPHPSVTMYVRLALVIIAGLFITVECSQEIIKNLSLQFAKPLEDCKKEMDLSDTVITDFYNFWKEGYEFTNRQFGCAILCLSSKLELLDQDLKLHHGKAQEFAKKHGADEAMAKQLVDMIHSCTQSTPDVADDPCMKTLNVAKCFVAKIHDLKWAPSMDLIMGEVLAEV*

>AipsPBP2

MAASRWCIACLVCVLFAARSVMTSQEVVASFSKGFTNVVEHCKAEVNAGEHIMQDIYNFWREEYQLVNRDLGCMVLCMANKLGLIGEDQKMHHAKAEEFAKSHGADEAVAKQLVAILYECETKHAAVEDECGMALEIAKCFRTKMHELKWAPSMEVAMEEIMTAV*

>AipsPBP3

MGTYNVFFAFVLMAAGVREIEPSKDAMKYITSGFVKVLEECKQELNMNDRIIADLFHYWKLDYTLLNRDTGCAIICMSKKLDLLDDTGRMHHGNAQEFALKHGAGEEVASKI VTIIHDCEKKFERDDDECLRVLEVAKCFRTGIHDLDWQPKVEVIVSEVFTDM*

>AipsGOBP1

MTQPGQVLVLVLLAAAALADVNVMKDVTLGFGQALDKCRQESDLTEEKMEEFFHFWRDDFKFEHRELGCAIQCMSRHFNLLTDSSRMHHVNTEEFIQSFPNGEVLARQMVALIHGCEKQFDHEDDHCWRILHVAECFKHACVAHGVAPSMEMMMTEFIMEAEAR*

>AipsGOBP2

MTLRCCLLLVVVAAVTRSVVGTAEVMSHVTAHFGKALEECRDESGLSAEVLEEFQHFWREDFEVVHRELGCAIICMSNKFSLLQDDSRMHHVNMHDYVKGFPNGEVLSGKLVELIHNCEKQYDTLTDDCDRVVKVAACFKVDAKAAGIAPEVAMIEAVMEKY*

>AipsOBP1

MDISKRRSKNAFRRLLVNTWLRLVQIFTCLSAPPVVSADVTSKCQGSKYENECDKLTCVFRKAKWLDGNAVDKAKLITYFEQFEKDHPEWAPAMQNVKTSCLGAELKTQGVFLNCPAYDVMHCVLGSFIKHATPTQWSTSASCSYPRAYAAACPICPEDCFSAQVPFGSCNACYLPPRTP*

>AipsOBP2

MSKFTCLVLCVVAASISRVHADDDANKAAFREAFKPILDECSKEHGVSNDDIDAAKKAGSADAIKPCFFGCIYKKAEVFNAKGEYDVDSALSKLKKFVPDEAKFAKYAEIGKKCASVNEKPVTDGDAGCERGAMLTACFLENRAEMLI*

>AipsOBP3

MIRSCRCLVFAAVFQVVLGQGLTGTDSGPPGFQRPQSYVPKHCFAPPPGVDLHTCCPIPQLFPDEDMESCGIQKLTKEQYENPSPARIPCQESICLLRNANLLKQNNSIDYEKMGDFVDNWAKMDPDFTIPITNAKKVCLIEGGPPAPPVCEPDRIFTCLTSYVLWNCKLRLDSGEGCKILKEHMDGCRPFLAGP*

>AipsOBP4

MFGYQFLSFAAALICFGSSYALTSEEEANIKEAFHPFIMKCAEEYGITEEQFEEAKEKHSAEGIDPCFMSCFMKESGFFDSAGKFDADKTKEFVDAHLTSERAITFMEAVGSECAKVNDEEVTDGDKGCDRAKLMWGCIQDLKEKMEGSE*

>AipsOBP5

MKYFVLFVALVAGIHANVTLPPEQSEKALKTASECIKETGVSKEVLAEAKKGHIADDEGLKKFTLCFFKKAGIVDNDGKLNLETALAKLPPGVDKAEAKKVLEGCQAKSGKTPQDTAFEIYKCYHAGAKTHIALAGI*

>AipsOBP6-PG

QRENKGASLKPLSVCCDIPELGDPKHLAKCSNPKLPGPCNDVQCVFEESGFLTDKNTLNKEAYRNHLKQWEENNKGWTVAVDKAIKECVDNDPRQHLDIPCKAYDVFTCTGIAMLKKCPDSAWKC*

>AipsOBP7

MSKFTCVLCVVALSLSSVYVTRAHKPNLRDAWRSELDECAKEYPVTNDEIDTAVRSGDSSNLNPCFNFCVFNKTGFFTENGEYDLKNGLIKLRKAIRDDEEYTKFEEVATECTEDKNTSCDEKAKCDSANRLSLCFLRFKDKVRI*

>AipsOBP8

MYLRSTNGGVRSFPLGESAYTTKIVEICSKETGLKKQVPPEEKEIKFSQRKGLREFNDCYLAKTGVTTSDGKLNIDEALEKLPPGFAKPFVEHCQANIILGYIEENVNDFSTCFHQEVQNHLLSFYGFENYWVMLVLGTSFDKTRFTTLFFDKHFDFWLAERAGFVNL*

>AipsOBP9

VFICGVLSLNVKASSLDELKMKYVEMIIECSDTYPITAADTLQLKTKTMPDNESIRCLFACVYKKAGMMNEQGELSVEGVNEMTRRYLSDDPDKIKKSEQFTEACKSVNDVPVSDGTRGCDRAALIFKCTVEKSPDFDLL*

>AipsOBP11

MTYKVFILVFLTYVSLATSALAPFITKCKWDDSKCIKESAQKVIPLFADGIPDLHVEKHDPLLIKRVDASSPNLKLIVTDIEVKGLKNCEAKKITRDLKAMKLSVKFLCAVDFKGVYDMKGQLFVLPIEGNGDLTAHVPKIQLNAEVDMVDKTGKDGKKHWGVKSWRHSFELKEKSNVKFENLFPDNEFLRKTTEELIASNGNDVIVEVGPEIIKAVTAKVIESIKKLFDEVPVEELAIDE*

>AipsOBP12

MYSGTIFLFSFILLIVSNVTFVSSQMTREQVKNSGKLVKKTCSAKNDLTEDEVKDVDKGKFIEEKKFMCYVACVYKMGQAVKGNSLNHDMMIRQVDMLFPADMKAPVKAAIE HCRPVAKKYKDICEASYWTAKCVYEFDPPNFMFP*

>AipsOBP13

MVLIYIVKFLILVAMCEAMTMKQIRNTGKMMRKSCQPKNNVEDEKIDPIAEGIFIDEPEVKCYMACIMKMANTLKNGKLNFDAALKQADLLLPDDIKEPAKEAIIACKKAAEGHKDICDVSFHVTKCIYNQNPGIFYFP*

>AipsOBP14

MFDPKTVFYLLTVFSVCFGAVDIRKYLKVCDRNAIDVSDCLTDAVQKGIAVMVNGIEELGVPPIDPYLQKEFRVEYNNNQIAVKMVIKNIYVEGLKDAKVHDARLRADDDKFHLEVDMTSPHVFVKAHYHGEGQFNSLKVVAYGDFNTTMSDLVYTWKLDGVPEKNGSETYVRIKEFYMRPDLSSIVTSFRNENPETRELTELGARFANENWRTLYKEFLPYAQANWNRIGVRIANKLFLKVPYDQLFPSSS*

>AipsOBP15

MDHNRLCLLVIAMFLATGSDAMTRQQLKNSGKILKKNCMNKHQVTEDQIGTIEKGKFVEDKKVMCYIACIYELTSVIKNNKLNYESSLRQIDIMYPADLKESAKAAVENCKDVQKKYKDICEASFHTAKCMYDFKPEDFIFA*

>AipsOBP16

MFPGSIPFISGCVHLGVSNYFRSTQSNLVVHYEDDQIVDAIYNCQDENGFDEVLSNSTNLEENFPEKEGLKKSNDCFLKKTGFVTSDGKLNIDKTLEKLPPSFVKPIVEHCQANIALNYTTESVENFSSCYHDGILNHIFAATEVGIFPFIQTWKFFVPGTSFADTILILN*

>AipsOBP17

MNQLLVFVLIVACVRISNGMTREQVKKTMTVIKKQCMPKNSVTEDQIGKIEQGVFNEDRNVMCYVACVYKSLQVVKNERLDLGLISKQIDALYPPELKEPTKKAVSQCINIQDSYNDLCEAVFHSVKCLYEKDPATFIFP*

>AipsOBP18

MKTLFVFAACILLAQALTDEQKEKLKKHRTECLTETKVEEALVNKLKGGDYKTESEPLKKYALCMMTKSELMTKDGKFKKDVALAKVPNAADKPSVEKLIDACLANKGNTPHQTAWNYVKCYHEKDPKHAIFL*

>AipsOBP19

MFTGTVPFVLCLVAVAFGGKDKPVFSEEIKEIIQTVHDECVANTGVAEEDITNCENGIFKEDPKLKCYMFCLMEEASLVDDDGTVDYDMLVSLIPDEYYERTTKMIFACKHLDTPDKDKCQRAFEVHRCSYEKDPDLYFLF*

>AipsOBP20

MLVINATDYDYEGYGTGNMGEKLLTSVPRPASSSNNINNNDTSRTRRSEPLLNKPDLDQCLSQCVFANLQVVDSRGIPREAELWNKVQSSVTSQQSRSALHDQIRACFQELQSEAEDNGCSYSNKLERCLMLRFSDRKVEGKASTPKPASTEQS*

>AipsOBP21

MLKFSVVCLYFSVAAVNFWNVHCISEDEKKAFIEAMKPMVEECGSDCGLTEEDYKKHSKGEDMDPCFKKCMMQKLGFLDEDGKYNRKQLHESISEYTGDKDEAKRVQEQLDSCFDANGDNDGDDEESQMKRVDVLFKCLKEIKE*

>AipsOBP22

MSMWFRAMVVVGALAAARCGVVMDEDMAELARMVRESCVDETGADVKLVEAVNGGADLMEDDKLKCYIKCTMETAGMMSDGEVDIEAVMALLPPEMAEHNGPALKSCGTQRGADDCDTAWKTQVCWQNANKAEYFLI*

>AipsOBP23

MSKFTYLVLCFVAVSRVYANEDERAAFHEAAKPILVECSKENGVSFDKLKAAKEAGSADGIDPCFFSCVFKKTGVFNSKGDFDLDNSLTKLKEFVSNDEDYAKVAEVGKKCE SVNEKDVSDGEAGCERASLLTACFLEHRAEIPV*

>AipsOBP24

MAKLLLAMILTVMTFALTMSATTKDAGTKEAIMTTTVANQDSSIDSNDVDVLAVMNVCNESFRIEMSYIQALNESGSFVDETDKTPKCFIRCVFENVGIVSEDGRMFNPARAAVIFAGERNGKPMDDIADMTALCAADRKETCPCDRSYQFLRCLMSMEIERYEKS*

>AipsOBP25

SRKLREAMRPIIEQCSKEHGVTDADIQASKDSNNAASLPDCFNHCLFEKSGFIDKNGRYDRDSGLKNLSKYLKDVNQYNKVVEVTKECASVEEKPATGCELGTRLTACLLDHQTSILI*

>AipsOBP26

MSKFTCIVLFVVAASLTKVTQAVSEEEKAVAREAMAPILAECSKAEGVSDEDIEEAKKNPSVDAVNSCFIRCVMRKTDALNEKGLFDSDAALAKIRPFVKSDEDFAKFEEIGKACMSVNDKEVSDGEAGCDRAKLLLACFLEHKAEMLY*

>AipsOBP27

DSAISADAESRCRNPPTAPQKIERVITLCQDEIKLSILREALDVIKEEHTMPAQRRRDKREVPFTHDEKRIAGCLLQCVYRKVKAVDGYGFPTLEGLVGLYSDGVNERGYFMAVLEASRECLMKNHDKFSRTMPMDNGRNCDVSFDIFECISDRIGEYCGTSGL*

>AipsOBP28

MTHIFSSFIPYMITVSMFSFPVSVKIISPTVPVTVVSSSVIITFMFDMYRLVILSIVAVTTVVADTDLQECRRLVHPHSMRCCKKSADAKEKMMKNDDLKECFDLPKDPVKCEHELCMAKKKGITTSDDKLDKAKFEEVVTKDIDDKDLVADIKANCINGDLTKYGPPDFCDFVKMRHCMSMQILNHCTEWNDFGDCPQLKSIIGDCVKLVAA*

>SlitOBP9

MCLVKYHVLVLCVILVGSYALNCRSSGGPKEAELKNIYKKCLKMQEGKNSSKGNSAQDWKEPRVQIQRNDWDRGRVGSKENKNSRDDSRSGSKDKKGDSGMRDNRNDMMSRRDDMMSRGDERNDNRKHRTDDRMGNDNDRSGNRGRGNKNNRNDMNGGRDDRFGRDDYFNGREDFPQSDEYGGDMGQYNNNYYSTTQSSRRYKRERRPSNSGQRSQYNPNNHKISGYEDNFRSDERNTTDNNSSKETDNKSCALHCFLENLEMTGEDGMPDRYLVTHAITKDVKNEDLRDFLQESIEECFQILDNENTEDKCEFSKNLLICLSEKGRANCDDWKDDLTF*

>SlitOBP10

MVRKISGLLCCLCVFGISFSDSAISADSESRCRNPPTAPQKIERVITLCQDEIKLSILREALDVIKEEHTMPAQRRRDKREVPFTHDEKRIAGCLLQCVYRKVKAVDGYGFPTLEGLVGLYSDGVNERGYFMAVLEASRECLMKNHDKFSRTVPMDNGRNCDISFDIFECISDRIGEYCGTSGL*

>SlitOBP11

MKSFVVFCIVFVVGVCATEKGNKIASECIKESGVKSDVLAEAKKGNLGDDPAFKEFTYCFFKKVGIVGEDGKLNRDVAIAKLPSGVDKAEAEKLLDSCKSKTGKDAVETVYEIFKCYQHGTKSHIMFAS*

>SlitOBP12

MKTLFVFAACILLAQALTDEQKEKLKKHRTECLTETKVDEELVNKLKGGDYKMDNEALKKYALCMMMKSELMTKDGKFKKDVALAKVPNPADKPTVEKLIDACLANKGNTPHQTAWNYVKCYHEKDPKHAIFL*

>SlitOBP13

MITSCLLVLSAVVQVLLAKQPVFESGPPEPWGPPERTSHPGQFQPRVPKRCWVPPQRINVYNCCPIPTLYPDEDMQSCGFEKLSENKPQKPVYRPEGTCKEGYCVMGKFDLLLANNSVDYVKFREYLDNWAESYPEFANAIHIAKEECAQDGGPEVPPICEPDKLFLCLTSTIFWNCKLRDGEGCAALQEHMNECKQYYTRVMAPTIKDFEVR*

>SlitOBP15

MYSINCFIFSVILIVMFDNCFVYSMTREQIKNSGKLIKKTCSAKNDLTEDEVKDVDKGKFIEKKDFMCYIACVYKMGQSVKGSTINHDMMLRQVDMMFPNDMKAPVKSAIEHCRPVAKNYKDLCEASYWTAKCIYDFDPANFMFP*

>SlitOBP16

MYRFVILSIVLVSALADDIDIRECGRIFHPPPHGCCKANNAVKNKDMLAEELKDCFDGSGPKDPMKCEIDLCIAKKKGFATDDGKLDIKKFEEVITKEVGSDKDLLDEIKTNCINGDLNNYGPPEFCDFMKIKHCVTLHMMNHCSEWSDDGNCKVVKELVGKCAKVI*

>SlitOBP17

MKTFRLLCCILSIFLFFDQSYGMTRQQLKNSGKLMKKSCMPKNDVTEDEVGDIEKGKFIETRNVMCYIACVYTMSQVVKNNKLSYEAVIKQVDVMFPAEMRDAVKAAATHCKETTKKYKDLCESSYWTAKCMYDYDAQNFVFP*

>SlitOBP18

MFKLCVFLALGFVACHGAPNSSPGTPNANPGTYCGVTPDNIYRCLNNPRVVTPEVSTKCGSQFTECEKMTCIFRELKWSKRGAIDKAKVRAYFDQYETEHPEWAQAVQHVKAFCLASELRAQGVFLNCPAYDIMQCVLASFIKHASPSVWSTATDCAYPKAYAADCPVCPSDCYSPQIPFGSCNACYTQPRTV*

>SlitOBP19

MFRRTLLLFSIIYISACNGQTEAPEKNRMMGIDAVHDNNVKIDKDTIITRNLKLEKRSRGPKSVSNKNEDQIEPDWSYANFPKEVSEHVEKFKKNMTECLKEVQTSDKRPVKRLSPKMESPVHGECLIACVLKRNGVIINGKVNKDNLIALVSKFYSKDTRLMKKLEKNLDRCIEMSVRAQDDCALALVLNDCTNDLMASNKHKIMVNY*

>SlitOBP20

MEKILIFTFITLSGFAHARISVMYAHDKLSDLVAQQCLSEMYPKNKRIEIQESDEPCIIFCVLKKFGIISASGVINLDIYRKRVQIAHQLDQKTSIMDYGGSCMENAEATQHKQDVCKKAKVFNDCTHLYRILLM*

>SlitOBP21

MARRQRGAMFTEALPLFVILVAVTHGGKNKPVFSDEIKEIIQTVHDECVAKTGVAEEDITNCENGIFKEDAKLKCYMFCLLEEASLVDDDDTVDYDMLVSLIPDEYYERTTKMIFACKHLDTPDKDRCQRAFEVHKCSYEKDPDLYFLF*

>SlitOBP22

MSKFTCIILCVVAASLTKVSHAAVTEEEKEAFREAMAPIIAECSEEHGVSEADIKAAKESASADNIKPCFLGCVMKKIEVLDAKGLYDAETGLGKLRKFVKDDDEFAKFEDIAKKCLKVNDESVSDGEAGCDRAKLVLGCFIEHKVEMPF*

>SlitOBP23

MAKFSCLVLCVVAASLGSIHVASGESLRESLRPVIVACSQEHGVTDAEIQAAKDAGSPASIKPCFIACVFKKAGFINEQGQLDLETGLKNLRQFVKDDEQYKKLEEVAKKCSQVKDKAVSDGAAGCERGVLLAGCFLEHKTSIII*

>SlitOBP25

MAKVTCIVLFVVGVSLSSIQADDGKNESEVEIDVNQIIDDCIEEYHIPRRLFLAAAETGSTHALTPCFWSCCFKGVGVLNSEGQYDIDATLDLSKKIFTDHEYEKVEIIVKKCESVNGAPVSNGNIECEKSVLLADCLFDNAKKHFPNMFGVDY*

>SlitOBP27

MYKFVILCSIFVAASNADVAQTLTKRETKASLKPLSVCCDIPELADEFQLAKCSPRPPGPCEDVQCIFEVSGFLTDRNTLNKAAYRSHLQKWEKNHPGWTDSIYKAITDCVDNDPRQHLEVPCKAYDVFTCTGIAMLKKCPDTAWKC*

>SlitOBP28

MIVRFLLCLYIVEFYGAHARTDQEIKAWFFREGMDCNIEHPISPKEMLELKENKIPDTNNAKCFVACVFKKTGMLDSKGMFDAENSIAMTQKDFANDPNRLESSKKLLEACKKVNDEAVSDGEKGCERSVLLHKCFVETAPQLGIKLP*

>SlitOBP29

MWNLLVVFLAICSCVYARRRSSGAEINGLTEEELKMEFTKLIMKCNKDGEVDMTELVQLQNYVVPTKQSTKCVLACAYKAAEVMNAKGEYDIDHAYKVAEMMKNGDEKRLVNAKKMADLCVKVNELSVSDGEKGCDRAAMIFKCTVENAPKFGFKL*

>SlitOBP33

MTCSQALALLALVAISQQATTGCKNCIMLGKEEKAMFRAHSDACVAASRVEPRLVDAMLAGELLDEPALRKHVYCVLLKCKLISKDGKLQKAAVLGKMAARPDAKNATKVLESCADQTGDTPEDLAWNLFRCGYDKKALLFDYMPTNVASETDNNS*
