# Supplementary material for: Identification and Comparative Study of Chemosensory Genes Related to Host Selection by Legs Transcriptome Analysis in the Tea Geometrid Ectropis obliqua
Source: PLoS One. 2016 Mar 1;11(3):e0149591. doi: 10.1371/journal.pone.0149591 (PMC4773006; doi:10.1371/journal.pone.0149591)
Supplement: S4 Table — (DOCX) [file pone.0149591.s005.docx]

>HarmCSP6

MKADCFLFVTLIAVVAADFYNSKYDSFDVQPLLENDRILLSYTKCFLDQGPCTPDAKDFKKVIPEALETTCGKCSPKQKQLIKTVIKAVISRHPDAWDQLTEKYDKDQKYKESFDKFLAEQD

>p10

MKCVAVFVIVAVVALAEAARFRRDDKYTTKYDNIDLDEILASDRLLANYHKCLIEEGKCTPDGEELKSHVSDALQNDCAKCSDKQRAGAEKVINFLYNKKKPMWESLQKKYDPENTYVTKYADRLKELHD

>EoblCSP1

MKVLILLTLVVLASARPDNHYDKKYDNFKVDELVSNSRLLKAYALCFLGRSKCTPEGHDIKRWIDEGTETRCAKCTPKQKVLVAKFIKALMEHCPEEWELLQKRSDPEGKQTDELKKFLDEFAP

>EoblCSP2

MKSFYILFAFFAVCAAQTVAPATSTSEAYYTSDENVDIEALVSNHDAMKQYVDCFTGKVECGPEAGAAKGEFPDALSDACAKCTQVQKHTSKVFFAEFKKSFPADYEAMKKAFDAENKLFPAFDAAIANA*

>EoblCSP3

MKGIVCVCLLAMAAAALARPDGDHYTDKYDNINVDEILENSRLRDPYLSCVLDVGKCAPEAKELKSHIKEALETHCLKCTDIQKEQTKKVIKYLINNREEDWKKLCDKYDPDRKYSKKYEDELKTVKA

>EoblCSP4

MKLVYLITLAGVAAIAYGKPAQYTDKWDNINVDQILESQRLLRGYVDCLLDRGRCTPDGKALKETLPDALEHECSKCTAKQKSGSDKVVRFLVNKQPALWKELGVKYDPNNIYQQRYKDQIESVKEKA

>EoblCSP5

MLVLSLLSVFSVALTQCEEIYTNKYDGVDLEEILANERLLTGYVNCLLDLGPCTPDAKELKKNLPDAIENDCEKCTDRQRDGADEVMHHIINHRPQDWQKLELKYNEDGSYKRKYLASKNTAASDEEKSTDTQDKNDKEDSEEKDESE

>EoblCSP6

MKFFYVFALSLVVACSGETYTTENDDLNIEAVVADLPTLQAFVGCFNDKVKCDEKSGDFKKDIAEAIQQACAKCTDAQKHIFKVFLTGLKEKLPADYAAFKAKYDPENKYFGNLEKAIANA

>EoblCSP7

MSVISVVSFVLIMTVVAQEKYYDRRYDYFNIDYFVNNPRLLKKYLNCFLDQGPCTPIGRVFKQVLPEVITTACKKCTPLQKRFTYKAFNAFKTMLPDLHQDLREKYDPQNKYYDEFEKVTSSA

>EoblCSP8

MKVAVVLCILSFAAVAFSAPSGGTYTSKYDGVDLDEILNNKRLLIPYVNCVLEEGRCTADGKELKSHIKEALETDCAKCTEAQQKGAEKVFRHLINHEPEIWDKLKTKYDPTGKYYKRHEAELKA

>EoblCSP9

MKLLLIAVLITLASLHTLAQTYNDKYDGMNIDQALANKRLLKAYINCVLDVGKCTPEGKELKGNISDALQSGCVKCTGPQRAGIRKVIAHLINHEERYWNQLVDKYDPKRLYSRKYENELKAIKG

>EoblCSP10

MRVIVVFACLVASVVTADKYSSKYDNFDVDTLINNDRLLNAYVNCFLDKGRCTPEGADFKKALPEAIETTCGKCSDKQKGNIRRVIKAIQQKHAKQWDDLVKKNDPTGNHRDNFEKFIQGS

>EoblCSP11

MRSSMLLCAVALWAFVSGDDVLSDEDELLIEDEKWFQGFHDCLMKTGPCPEEIKKYTNIIPIYLKTACGPCSPKEKEIFQKHKQLVAAIYPKMFADLKAKFVTNTTKATENL

>EoblCSP12

MKWTVLAVCALTVVVSSQQYPNRYENFNTDAIIQNERILLAYYKCVMDKGPCTKDGKNFKRVLPETLTTACARCSPKQKQVVRKMLLGIRAKSEPRFFELLDKYDPEQTNREALYKFLVTGF

>EoblCSP13

MKSFCIVALGLVAACNAAYTSDAGLDVDTIVKDKEAVQKVLKCFNGGDCSSQAAALKTDFPLALGSACAECTPEQKHNTKLFIGAVEKFFPEEYKSLKTNFDPENKLFPGLLAAVAAH

>EoblCSP14

MQIKYATILSALIAVCIAQSQRPQVSDTALEDALNDKRFIQRQLKCALGEAPCDPIGKRLKTLAPLVLRGACPQCSPQETKQIQKTLSYVQRNFPQQWAKIVRQYAG

>EoblCSP15

MNALLLLAIVTLAVPLVVCYDEIYDKIDVDKIIADDALFTKYIDCMLDKGPCEVEHSADFKKLLPEVIATSCAKCSPIQKKHVRKTVKTLGEKKPAELLEFKKMYDPKGEHEKDFTAFVLAED

>EoblCSP16

MKTVLVFCVIVGAVLCAPQEHYSTQYDNFDAQELAQNPRLLKNYAKCFLSQGPCTSEGTDFKKVIPDAVKTKCEKCSDKQKELIRIVVKALQEKLPELWQELVKKEDPNGQYKAAFESFITGN

>EoblCSP17

MLKMKFFLVICCLAAAVVAEKYSDKFDNIDLQEILENKRLLKAYVDCVMERGKCSPEGKELKEHLQDAIETGCEKCTEKQEKGSYTVLEHLIKNELEIWRELTAKFDPEGKWRKKYEDRARANGITIPEE

>EoblCSP18

MKVISFYMVMMMVMVAYANPEEKYREPNDVNIEEIIKTPRLFHGYYLCLSKEGKCTPYGKELKENMPDALANGCAKCTENHMTAIRKVIKFMIENKPEEWKKLKSIYDPEGIYAAKYEKELKELQA

>EoblCSP19

MRAVITLGVCLCVCVVMCQDLNDMSQMPKYDSRYDYLDVDDIFTNKRLVRIYVDCLTSSVRCTPEGKALKRLLPEALRTKCVRCTERQKRTAVKIIKRLKYEYPDEWAKVSTRWDPTGDFTRYFEEFLAKEQFNSIPGSGVVLPLSTVTQQPPPPPAPVPAPAPAPAPSTNPPPTAAVLPLSTSPRPIILNRFGGDGELMIGSPSSAAGTTRPATPPGVKISTMRPLMQTRPTPAASSGSASNAIPTRFPLRPTTDLPLPYSTAITLIDQIGYKIIRTTELVTDLLRNTVRAVVG

>EoblCSP20

MKFAIIALCLVAAVLASDKYDELNDNFDISEVLNNPRLLNSYAKCLLNRGPCTPEVKQVKEKLPEALETRCAKCTEKQKQMGKQLAQEVKKHHPKLWSDLVALYDPEGKYQQAFQDFLASQ

>EoblCSP21

MKTATVLCVFAIVALAAARPDAGKYTDRYDNVNLDEVIGNKRLLVPYINCVLEKGRCSPDGKELKSHIKEALETYCAKCTDVQRSGTRRVIGHLINHEPGYWAQLTNKYDPARKYVVRYENELRQTQG

>HarmCSP

MKVLLVLCLFAAAALADDKYTDKYDNINLDEILENKRLLLAYVNCVMERGKCSPEGKELKEHLQDAIETGCSKCTEAQEKGAYKVIEHLIKNELDIWRELAAKYDPKGDWRKKYEDRARANGIQIPE

>HarmCSP12

MNSAIVLCVVALAGMVLARPDGDGDKYTSKWDNIDLDEILGNDRLLVPYIKCALDEGKCAPDAKELKEHILEALETGCDKCTDKQKEGTHRVIAHLIKYKLEEWEKLRAKYDPEGKYAKKYEKELEELKRA

>HarmCSP11

MNSAIVLCVVALAGMVLARPDGGTYTTKYDNVDLDEILANDRLLIPYIKCLLDEGKCAPDAKELKEHIREALENGCAKCTDKQKEGTRRVIAHLIKHKNADWQKLKAKYDPEGKYTHKYEKELEEVQH

>HarmCSP10

MKVLVVLSCLIVAAFAADKYNAKYDNFDVDTLITNDRLLKAYINCFLDKGRCTPEGSDFKKTLPEAIETTCGKCTDKQKNNIRKVIKAIQQKHPKEWDALVKKNDPSGKHRANFDKFIQGSR

>HarmCSP9

MNSLIVFCVLSLAALTIARPDGATYTDKYDNVDLDEILGNRRLMVPYIKCMLDQGKCAPDAKELKEHIKEALENECGKCTEAQKKGTRRVIGHLINHEADFWNELAAKYDPERKYTTKYEKELKEVEA

>HarmCSP8

MKCIYVLSFLLALAAVQAEDKYSTENDNLDIDAVVANVDTLTSFVACFVDQEPCDAVAADFKKDIQEAVTTRCAKCTDAQKHIFYKFILGLKEELPRGYEEFGRKYDPENKHFSALENAVSPA

>HarmCSP14

MNSAIVLCVVALAGMVLARPDGDGDKYTSRWDDVDLDEILENDHLLIPYIKCSLDEGKCAPDAKELKEHIQEALETGCAKCTDKQKEGTRRVIAHLIKKKLQEWEKLKAKYDPEGKYAKKYEKELEEVKNA

>HarmCSP13

MKVLLVLCLFAAAALADDKYTDKYDNINLDEILENKRLLLAYVNCVMERGKCSPEGKELKEHLQDAIETGRSKCTEAQEKGAYKVIEHLIKNELDIWRELAAKYDPKGDWRKKYEDRARANGIQIPE

>HarmCSP15

RPESQYTNKYDNVNLDEILVNKRLLVPYIKCALDQGKCSPDGRELKSHIREALENYCAKCTPVQQDGTRRVIAHLINHEPDYWRQLSVKYDRDGKFAVKYEKELRTIA

>HarmCSP16

MKILVLLLAAVVTAQYEEDTYGTDHDDLDIVALVEDKDQFNSFIDCFIDEAPCDDVAETFKSVIPEAVLEVCAKCTPAQKHIVRVFNESFKKKMPEKFQKFKNKYDPEGKYFENFEAAVGAF

>HarmCSP17

ASTYTDKWDNINVDEILESQRLLKAYVDCLLDRGRCTPDGKALKETLPDALENECSKCTDKQKSGSDKVIRHLVNKRPEMWKELSAKYDPNNIYQDRYKDKIEAVKGQ

>HarmCSP1

MRAVLFVCALVYAVAAQDVSDMVNMPKYDSRYDYLDVDAVFTNKRLVRNYVDCLINAVRCTPEGKALKRILPEALRTKCVRCTERQKRTAVKVIKRLKNEYPDEWAKLASRWDPTGDFTRYFEEFLAKEQFNTIPGSAGIGSEIPTSSPLAPPRAPTVAPTVATPTAAATEPTPPRPVVLNRFGDEGELMMGSPSSAGITPRPMTQATTRPTTTMRPVNTRPVPPRPTMMTWAGAASNTQPTRFPLRPSPSDVPPPYSTAITLIDQIGYKIIKTTELVTDLLRNTVRAVVGR

>HarmCSP2

MKTAIVLLLALFGVVLTARFDNIDIDKVLGNQRVLESYLKCMYDEGPCTPEGRDLREKAPEALETNCKDCTDNQKALVRKASLFLIKNRPDDWKKLSDKFDPEGKYKKAFDEFLKEKN

>HarmCSP3

MKAVFLLCLVVVAVSARPEAQYTNKYDNVNLDEILVNKRLLVPYIKCALDQGKCSPDGRELKSHIREALENYCAKCTPVQQDGTRRVIAHLIKHKLEEWEKLKAKYDPEGKYTHKYEKELEEVQH

>HarmCSP4

MQTRYAVVLCCVVAACVAQTQRPPVSDSALEDALQDKRFIQRQLKCALGEAPCDPIGKRLKTLAPLVLRGACPQCTPQETKQIQRTLSYVQRNFPQQWAKIVRQYAG

>HarmCSP5

MKSLLLLCLVIAAVWARPETYDTRYDDFDAETLVENVRLLKAYGHCFLGTGPCTPEGSDFKKTIPDALRTGCGKCTAKQRHLIRVVVQGFRSKTPDLWQQLVKKEDPNGQYKEVFTRFLNGSD

>HarmCSP6a

MKLIVAVALLCLVAESWAASTYTDKWDNINVDEILESQRLLKAYVDCLLDRGRCTPDGKALKETLPDALENECSKCTDKQKSGSDKVIRHLVNKRPEMWKELSAKYDPNNIYQDRYKDKIEAVKGQ

>HarmCSP7

MNSLIVFCVLSLAALTIARPDGATYTDKYDNVDLDEILGNRRLMVPYIKCMLDQGKCAPDAKELKEHIREALENGCAKCTDKQKEGTRRVIAHLIKHKNADWQKLKAKYDPEGKYTHKYEKELEEVQH

>AipsCSP8

MNFLVLSMVIALAGFVAAETYTDRYDHINIDEIIENRKLLVPYIKCTLDQGRCTPEGRELKAHIKDAMQTSCSKCTPKQRKGARKVVKHIRAKEQEYWNQILAKYDPENQYSENYEAFLAADD

>AipsCSP7

MKFVLLLCVMVAVVYAEDKYTDKFDNIDLDEILTNRRLLLSYFNCVMGKGKCTAEGKELKDNLEDAIKTGCAKCTENQEKGSYRVIEHLIKNELDLWRELCAKFDPTGEWRQKYEDRARANGIEIPKD

>AipsCSP6

MKLIIAVALLCMVAASWGKPASTYTDKWDNINVDEILESQRLLKAYVDCLMDRGRCTPDGKALKETLPDALENECSKCTEKQKSGSDKVIRHLVNKRPDLWKELSTKYDPDNIYQDKYKTQIESVKQ

>AipsCSP5

MQIKYALLLCCVAAMSVAQTQRPAVSDTALEDALQDKRFIQRQLKCALGEAPCDPIGKRLKTLAPLVLRGACPQCTPQETKQIQRTLSYVQRNFPQQWAKIVRQYAG

>AipsCSP4

MKVVLLTLCFALGVLAQDKYESVNDDFDVSKVLNNDRLLQSYAKCLLNKGPCTSEVKEVKAKLPEALETRCAKCTDKQKQMGKVLAQEVKKNHPDIWKELVAMYDPQGKYQEAWKEFLQE

>AipsCSP3

MNSFIVLCIASLAVMAYARPEEAKYTDRYDNVDLDEVLSNRRLLVPYVKCILDQGKCAPDGKELKEHIREALENECGKCTETQRKGTRRVIAHLINNEADYWNELTVKYDPQRKFTAKYEKELKEIKQ

>AipsCSP1

MKAVIVLCALVVAVCARPEEEKYPDKYDNTNYKEILENGRLYRAYCDCLLDAGKCTPEGKELKSRIKDALETKCEKCTDKQKEAVRYVIKYLINKKPEDWKKVCDKYDPDGKLKSQYEKELKDL

>SexiCSP20

MQIKYALVLCCVAAVSVAQSQRPPVSDTALDDALQDKRFIQRQLKCALGEGPCDPIGKRLKTLAPLVLRGACPQCTPQETKQIQRTLSYVQRNYPQQWAKIVRQYAG

>SexiCSP19

MKYILVALVATIAVVKAQETYGTQYDNVNGEAIVSDDQQFQSFVDCFMGAATCNEPAAAFKKVLPEAIVQACAKCNPAQKHLVRVFLEAYSKKMPQEYEKFKDLFDPERKYFPKFEASVAGF

>SexiCSP18

MKGITMICALGVLACAVASPADHYTDRFDNINIDDILNNPRLLNAYINCVLDKGKCTSEGKELKSHISDALENHCEKCTEKQRQGTRTVLAYLINNKPATWNQLTAKSIPMEICRSV

>SexiCSP16

MNALLIAVFALAAPLVLGYDEKYDKLDVDKILGDDALFTAYIDCMLDKGPCSVEHSADFRQLLPEVISTACAKCSAIQRQNVRKTVKALSEKKPDDFAQFRTKFDPKGEYEKAFSAFVIGTD

>SexiCSP14

MRAVLFLCALVHVVVGQDVNDMVNMPKYDQRYDYLDVDAIFANKRLVRNYVDCLINAVRCTPEGKALKRILPEALRTKCVRCTERQKRTAVKVIKRLKNEYPDEWSKLASRWDPTGDFTRYFEEFLAKEHYNTIPGSGSALPTSAPIAPPRVSPLPPSTTPTPGPTESTPPRPLVLNRFGDDGELMMGSPSSAGVTPRPMTQATTRPSTTTKTPSTRPIPPRPTMMTWAGAASNTQSTRFPLRPVSEISPPYSTAITLIDQIGYKIIKTTELVTDTLRNTVRAVVGR

>SexiCSP13

MKSILVLCLLVTAVSCRPETYDTRYDNFDVEALVGNVRLLTAYGHCFLGTGPCTPEGSDFKKTIPDALRTGCGKCSPKQRHLIRVVVQGFQNKTPALWQQLVKKEDPNGEYKEIFTRFLNAKD

>SexiCSP12

MKLVIILALVAIALARPDDGFYDKKYDNFNADELIENDRLLKSYAHCFLEDGKCTPEGNDFKKWIPEATTTSCGKCTDKQKVLVAKTIKAIKEKLPAEYEALVKKHDPEHKHHDDLNKFLEKYAP

>SexiCSP11

MRVLVVLSCLVVVAFAADKYNPKYDNFDVDTLISNDRLLKAYINCFLEKGRCTPEGSDFKKALPEAIETTCAKCTDKQKGNIRKVIKAIQQKHPKEWEDLVKKNDPSGKHRGNFDKFIQGSS

>SexiCSP10

MRSWLLCLCVLTVVVSCYSQANRYENFNPDAIVQNDRILLAYYKCVMDKGPCTRDGKNFKRVLPETLATACGRCNPKQKTIVRKLLLGIRSKSEPRFLELLDKYNPDRSNRDALYAFLVTGA

>SexiCSP8

MQIVVVLVVACVGLVAGLHVQAGPQMTDAQLEQTLADKSTMQRHIKCALGEGPCDPVGRRLRTLAPLVLRGACPQCSMQETRQIRRTLAFVQRNYPWEWAKIVRQYG

>SexiCSP7

MKFVLVLCLMAAAVLADDEKYTSKYDNIDLDEILTNKRLLTAYVNCIMERGKCSPEGKELKEHLVDAIETGCTKCTENQEKGAYKVIEHLIKNELDIWRELTGKYDPSGKWRKTYEDRAKANGIIIPE

>SexiCSP6

MKLIVVVALCLVAVAWAKPASTYTDKWDNINVDEILESQRLLKAYVDCLLDRGRCTPDGKALKETLPDALEHECSKCTEKQKKSSDKVIRHLVNKRPDLWQELSGKYDPENIYQERYKNQLDAVKRQ

>SexiCSP4

MKCIYVLSVLLAFAAVQAEDKYSTENDDLDIEAVVADLDTLKGFVGCFMDAMTCHAVAADFKKDIPDAVATSCAKCTNAQKHIFHKFLLGLKEKLPSDYEAFKKKFDPQGQYFEALEAAVASS

>SexiCSP5

MIGLNKYNVPVSIILIFLFVSTVLSQEKFYDRRYDYYEIDTLIQNPRLLKKYLDCFLGKGPCTPIGRVFRQILPEAVQTACKKCTPSQRRLARKTFNAFKGYFPETHEELRKKLDPKNKYYEAFEKAISSA

>SexiCSP3

MKVVFLVFVLTAVVYSHPHDSHYTDKYDNIDLDEILNNKKILTSYINCCLDLGKCTPDGKELKSHIREALENKCGKCTEAQKNGTRKVMTHLINFEPDYWNQLCAKYDPEGKYKAMYEKEYKTLVH

>SexiCSP2

MKSMIVLCVLSVAALVVARPDDSHYTDRYDNVDLDEILSNRRLLVPYIKCILDQGKCAPDAKELKEHIREALENECGKCTETQKKGTRRVIEYLINNEEEYWNELTAKYDPERKYTTKYEKELKKIKA

>SexiCSP1

MKSFIVLCLFGLAAVAMARPDGSTYTDRYDNINLDEILGNRRLLTPYIKCILEEGKCTPDGKELKSHIREALEQNCAKCTDAQRNGTRRVLGHLINNEEESWNRLKAKYDPQSKYTVKYELELRKLKQ

>BmorCSP16

MIEWKRFKILHFLSYLGLLVLVVVCAAQQNRPQVTDTALDEALNDKRFIQRQLKCALGEAPCDPIGKRLKTLAPLVLRGACPQCSPQETKQIQKTLSYVQRNFPQHWAKLVRQYAG

>BmorCSP15

MIENFYSKCTISKSVLFLCLIFLPYALNQKYYDSRYDYYDIDHLVQNPRLLKKYLDCFLGKGPCTPIGRLFKQVMPEVITTACAKCTPTQKRFARKTFNAFRRYFPETLMELRRKFDPESKYYDAFEKVITNA

>BmorCSP14

MKSSLFCVLVLTVVVSSSRQQSYPRNDNININAILQNDRILLGYFKCVMDRGPCTKDGKTFKRALPEALPTACARCSNKQKAAFRTLLLAIRARSEPSFLELLDKYDPSRSNRELLYTFLATGL

>BmorCSP11

MKLTSFLLVGMAMVSAEFYSSRYDDFDVKPLVENDRILQSYTNCFLDKGPCTPDAKEFKKVIPEALETTCGKCSPKQKQLIKTVIKAVIERHPEAWEELVNKYDKDRKFRPSFDKFINEDD

>BmorCSP12

MFMLFIISFIIVPVLKCCGTETSTYTTQYDEVDIKEIMGNERLLVAYIGCLLDKNPCTPEGKELKRNIPDALQSDCSKCSDKQRENADAWIEFMIDNRPEDWTKLEER

>BmorCSP13

MKLLLVFLGLFLAVLAQDKYEPIDDSFDASEVLSNERLLKSYTKCLLNQGPCTAELKKIKDKIPEALETHCAKCTDKQKQMAKQLAQGIKKTHPELWDEFITFYDPQGKYQTSFKDFLES

>BmorCSP10

MKILIIVVMACVAVTWARPESTYTDKWDNINVDEILESNRLLKGYVDCLLGKGRCTPDGKALKETLPDALEHECVKCTGKQKSGADKVIRHLVNKRPDLWKELAVKYDPDNIYQARYKDKID

>BmorCSP9

MRAVIFLYTCVFVVVGQDINAMMSMPKYDERYDYLDVDDIFRNKRLVRNYVDCLINAQRCTPEGKALKRILPEALRTKCIRCTERQKRTSVKVIRRLKNEYPEEWAKLASRWDPTGDFTRYFEDYLAKEHFNTIPGSGPTVNVLSLQTTPPPPPPPPSRPASVFTNPPPPVMSTSPRPVVLNRFRR

>BmorCSP8

MKTILILCALVSVVVCRPEEYYSSQYDNFDVEQLVGNLRLLKNYAKCFLDQGPCTAEGTEFKKRIPEALRTKCAKCNPKQRHLIRTVVKAFQTKLPDLWEELAIKEDPKGQYKHEFTAFINAMD

>BmorCSP4

MKVLIVLSCVLVAVLADDKYTDKYDKINLQEILENKRLLESYMDCVLGKGKCTPEGKELKDHLQEALETGCEKCTEAQEKGAETSIDYLIKNELEIWKELTAHFDPDGKWRKKYEDRAKAKGIVIPE

>BmorCSP5

MKTVIVCLLALTAVALARPEQYTDKYDTVDLDQLISNRRLLIPYVHCILEKGQCTAEGKELKSHIKEALETNCAKCTKAQKGGTEKMIGHLINHEAEFWEELKAKYDPTNEFTKKYETELKRVTA

>BmorCSP6

MKSLIVLSCLLAACLAADLSKYENFDVEPIVTSDRLLKAYINCFLDKGRCTPEASDFKKALPDTIATNCGKCTEKQKANVRKVIKVIQQKHSTEWEKLVKKHDPSGKHRADFDKFLLGS

>BmorCSP7

MKGFYVLCFALFAAVYCKETYSSENDDLDIEALVGNIDSLKAFIGCFLETSPCDAVSGDFKKDIPEAVAEACGKCTPAQKHLFKRFLEVVKDKLPQEYEAFKTKYDPQGKHFDALLSAVANS

>BmorCSP3

MNSLIAFCLFAVLAVALARPDDKYTDRYDNVNLDEVLSNSRLLQPYIKCILDKDRCAPDAKELKEHIREALETECAKCTEAQKKGTRRVIGHLINNESKSWNELTAKYDPENKFTAKYEKELREIKA

>BmorCSP2

MKSVILICFLGVATVVIARPKTPFDNINIEEIFENRRLLLGYINCILERGNCTRAGKDLKSSLKNVLEENCDKCSEDQRKSIIKVINYLVSSEPESWNQLKSKYDPEGKYLIKYEAKMESN

>BmorCSP1

MKCLTIAALLFVAGLSIAEKYTDKYDNIDVDEILENRKLLVPYIKCVLDEGRCTPDGKELKAHIKDGMQTACAKCTDKQKVSARKIVKHIKQHEADYWEQMKAKYDPKDEFKEIYEGFLAGQN

>TcasCSP8

MIFKIHFLVFGALLTYVSSVEYLILREIDTILKNDQMTRNYLDCVLDKGKCTKEAEKLKKGITETMKNGCVKCEQKQKEDVHKVFQHLMIHRPNWWHELETKFNPHHEIKLQHLHQSKFNPHEEVKLQHLHQFPHHDFLEREGFIR

>TcasCSP19

MKFFIAFLMLLGAVWCEQYTTKYDNINVDEILASERLLKNYFNCIMDRGACTPDADELKRVLPDALKSDCAKCSEKQKEMTKKVIHFLSHNKQQMWKELTAKYDPDGIYFEKYKDKFDS

>TcasCSP18

MRFFVIFFVACVSVALARPEDQYTIKYDNVNLKEILQSDRLTENYVNCLLEKKPCTPDGEELKRVLPDALKTSCAKCTDKQKQGAKTVIQHLYKNKQDWWKQLEAKYDPEHTYVKAHEDELKAL

>TcasCSP16

MPLVKSLVVVVLLIGVVYQVQGQLGLAGNNYIEKQLLCALDKAPCDALGNQIKGALPEIIGKNCERCDSRQVANARRIARYVQTKHPDVWNALVKKYSV

>TcasCSP12

MKLISAVILCAFLVAVSAAENKYTNKYDNVDVDKILNNDRVLTNYIKCLMDEGPCTSEGRELKKTLPDALSSGCTKCNQKQKETAEKVIRHLTQKRARDWERLSKKYDPQGQYKKRYEEHVATSRAA

>TcasCSP13

MIPLIAIAGILAVSAAPAEFYESRYDHLDVESILNNRRMVNYYAACLLSKGPCPPQGVDLKRVLPEALQTNCAKCTEKQRTAAYRSIKRLKKEYPKIWEQLRAVWDPDDVFIRKFETSFESGKPSGVISTNTSPPSPILSNRFGENEEADAASNVISSTPLPPTTSTTTRTTLTTKFTTKPSTKPTNKPVVVTKPPQAPPFATVGANLQATVSFGTNLVGGIVRSLGTLGSRVVESGTKLANMVISAAIRP

>TcasCSP14

MIKRKFRFIPVHDFVNGKTLHRSTRDDKYTTRYDNVDVDRILHSKRLLLNYINCLLEKGPCSPEGRELKKILPDALVTNCSKCSEVQKKQAGKILTFVLLNYRNEWNQLVAKYDPDGIYRKQYEIDDDYDYSELDSAKK

>TcasCSP11

MYSYLIPLYLFLFVHYGWSEDTTHKYTTKYDNIDLENVVKNERLLKSYVDCLLEKGRCSPDGLELKKNMPDAIETDCSKCSEKQKEGSDFIMRYLIDNKPDYWKALEAKYDPDGTYKKRYFESQKDEVSKVEA

>TcasCSP10

MKLFVINFILMSLVYMSFGASVPYETVDIDKLLADDKMVTEYMACLRGEGPCNPAEKDLEEHIPLVLGNYCADCNDKQKNFVIKLATFVIKNRFDEWRQVQKRFDPDLSHADDFNKFILGS

>TcasCSP7

MKTFVLVAFAAVLGLALARPQEKYTTKYDNIDLEEILKSDRLLKNYFNCLMERGTCSPDGEELKKALPDALHSGCSKCTEKQKEGSRKIIHYLIDNKRDWWNELEAKYDKDGVYRQKYKDVIEKEGIKL

>TcasCSP9

MKTLVLVLFVAVLSVVFAADKYTTKYDNIDLNQILKSDRLLKNYVNCLLDRGKCSPDGQELKNNLADALQTSCSKCSQRQKDGSRTIIRYLIKNKRDWWNELEAKYDPTGIYKNKYADELKAEGIVL

>TcasCSP1

MKTLVPLLFFVIAIASSLAENSKYTTKYDNVDLDEIIKSDRLLKNYVNCLLEKGKCTPDGAELKRHLPDALHTECSKCSETQKNGSKKIMRHLIDHKRDWWNELEEKYDKEGEYRKKYEAEIKGKKD

>TcasCSP6

MFLAIVLVVCACTNVLSEEYTNQYNDELDAALKSERLMKSYFECLLGTGKCTPSGEELKKDIPDALKNECAKCNDKHKEGIRKVIHYLVKQKPEWWEQLQKKFDPQGIYKKRYQNYLDKEGLKA

>TcasCSP2

MFATSALFAFICIQGLVSAEEYLVPQNIDLDEILKNDRLTRNYIDCILGKGKCTPEGEELKRDIPEALQNECAKCNEKHKEGVRKVLHHLIKNKPNWWQELEAKFDPKGEYKQKYNKLLEKEGLQA

>TcasCSP4

MFKVLFVVFACVQAYVYAEEYTVPQNIDIDEILKNDRLTKNYLDCILEKGKCTPEGEELKKDIPDALQNECAKCNEKHKEGVRKVIRHLIKNKPSWWQELQEKYDPKGEYKSRYNHFLEEEGLN

>TcasCSP5

MTAIVFLLALACLKTYVSSQEYLVPQNIDVDEILKNDRLTRNYLDCVLGKGKCTPEGEELKKDIPEALQNGCAKCNEKHKEGVRKVIHHLIENKPNWWQELESKFDPQGEYKKKYDELLKKEGLAN

>TcasCSP3

MLFTVFLVLTCAHVVFLEEYVIPDNIDIDDILSNERLLKNYVNCLLDKGRCTPEGKKLKSTIPEALSTDCAKCNEKVKANVRKVLHHLIDNKPDMWKQLEAKYDPSGEYRSKYKDELEKNGIHV

>TcasCSP20

MKIIILAVLIATAVAATYDVYPTKYDNVDIDAILHNKRLFDNYLQCLLKKGKCNEEAAILRDVIPDALITGCRKCNDHQKVSVEKVIRFLIKERNSDWQQLISVYDPKGEYQTQYAHYLEKI

>TcasCSP17

MHCLLQFCLLAAIFTCVKPQLTRISDEAIESTLNDRRYLLRQLKCATGEAPCDPVGRRLKSLAPLVLRGSCPQCTPQEMKQIQKVLAFVQKNYPKEWNKILHQYAG

>TcasCSP6a

MMGSKGPTGLHGPITSTGACFAGRYSPTYRSPEPMARRCMPNPSSRILEDASIMCNSWSPRHNGDLFSGLNDGLISRAEALAAVDIKHQSSGGPGGLPQLKHDMMYHHSMGAPPPVSRPHQMGHMDGLEMLDPISTSSMTTLTPMSETSSHMHSYGMNHVMNHHHHGGPVAPHPGHHGGHPGAHHPAMAAAAAAAAVAGLHPDADTDPRELEAFAERFKQRRIKLGVTQADVGKALANLKLPGVGALSQSTICRFESLTLSHNNMIALKPILQAWLEEAEAQAKNKRRDPDAPSVLPAGEKKRKRTSIAAPEKRSLEAYFAVQPRPSGEKIAAIAEKLDLKKNVVRVWFC

NQRQKQKRMKFAAQH
